# Transferrin receptor 1 levels at the cell surface influence the susceptibility of newborn piglets to PEDV infection

**DOI:** 10.1371/journal.ppat.1008682

**Published:** 2020-07-30

**Authors:** Shuai Zhang, Yanan Cao, Qian Yang

**Affiliations:** MOE Joint International Research Laboratory of Animal Health and Food Safety, College of Veterinary Medicine, Nanjing Agricultural University, Weigang, Nanjing, Jiangsu, PR China; University of North Carolina at Chapel Hill, UNITED STATES

## Abstract

Porcine epidemic diarrhea virus (PEDV) mainly infects the intestinal epithelial cells of newborn piglets causing acute, severe atrophic enteritis. The underlying mechanisms of PEDV infection and the reasons why newborn piglets are more susceptible than older pigs remain incompletely understood. Iron deficiency is common in newborn piglets. Here we found that high levels of transferrin receptor 1 (TfR1) distributed in the apical tissue of the intestinal villi of newborns, and intracellular iron levels influence the susceptibility of newborn piglets to PEDV. We show that iron deficiency induced by deferoxamine (DFO, an iron chelating agent) promotes PEDV infection while iron accumulation induced by ferric ammonium citrate (FAC, an iron supplement) impairs PEDV infection *in vitro* and *in vivo*. Besides, PEDV infection was inhibited by occluding TfR1 with antibodies or decreasing TfR1 expression. Additionally, PEDV infection was increased in PEDV-resistant Caco-2 and HEK 293T cells over-expressed porcine TfR1. Mechanistically, the PEDV S1 protein interacts with the extracellular region of TfR1 during PEDV entry, promotes TfR1 re-localization and clustering, then activates TfR1 tyrosine phosphorylation mediated by Src kinase, and heightens the internalization of TfR1, thereby promoting PEDV entry. Taken together, these data suggest that the higher expression of TfR1 in the apical tissue of the intestinal villi caused by iron deficiency, accounts for newborn piglets being acutely susceptible to PEDV.

## Author summary

Newborn piglets are particularly susceptible to infection by PEDV, with 80–100% dying within days of infection. The reasons for newborns’ acute susceptibility to PEDV infection have not been elucidated clearly. The primarily target of PEDV is the porcine intestinal epithelial cells. Here, we show that the high expression of TfR1 in the apical tissue of intestinal villi in newborn piglets with iron deficiency is a reason for their susceptibility to PEDV. Further, we demonstrate that iron supplementation reduces PEDV infection. This study reveals that iron plays an important role in the susceptibility of newborn piglets to PEDV and provides insights into therapies for the prevention and treatment of PEDV infections.

## Introduction

The newborn piglets are at risk of iron deficiency; they are born with low iron reserves, the concentration of iron in sows’ milk is low, and the lack on contact with soil in modern confinement rearing systems combine to create conditions insufficient to meet the requirements for rapid growth and increase in blood volume during this time [1,2]. Iron supplementation of newborn piglets is essential, and anemia in newborn piglets occurs, almost without exception, if none is provided during the first few days after farrowing [3]. Iron deficiency in the newborn pig population is often comorbid with a viral infection. Iron deficiency is associated with an array of diseases when iron-requiring enzymes become ineffective [4]. As an example, many HIV-positive individuals suffer from iron deficiency [5] which hinders their ability to limit infection, especially when the virus attacks immune cells [6,7]. Iron-loaded transferrin binds with transferrin receptor 1 (TfR1) on the surface of cells, which then delivers the iron into the cell [8]. TfR1 mediates cellular iron uptake, and as such, is a key molecule in cellular iron homeostasis and plays a central part in the homeostasis of the intestine [9,10]. Generally, TfR1 expression is regulated by iron levels and reflects the metabolic demands for iron; TfR1 expression levels increase under the conditions of iron deficiency [11]. Low iron levels trigger iron regulatory proteins to bind to the iron-responsive elements in the 3′-untranslated region of TfR1 mRNA, thereby preventing its degradation and enhancing its translation through the iron-responsive element/iron regulatory protein system [12,13]. A variety of viruses can utilize TfR1 for binding and entry into host cells, including New World hemorrhagic fever arenaviruses, mouse mammary tumor virus, Machupo virus, canine and feline parvoviruses [14–17].

Porcine epidemic diarrhea virus (PEDV), a member of the *Coronaviridae* family, can cause acute, severe atrophic enteritis, including mild to severe watery diarrhea, dehydration, and vomiting in pigs. Newborn piglets are particularly susceptible to PEDV infection and suffer a fatality rate of 80–100% [18,19]. PEDV contains a glycosylated peplomer protein (spike, S), an envelope protein (E), a glycosylated membrane protein (M), and an unglycosylated RNA-binding nucleocapsid protein (N) [20–22]. Virus entry is the initial step of viral infection and PEDV entry is mediated by trimers of the large S glycoprotein which attach to specific host receptors. The species-specific virus host-range or tropism is usually determined by entry receptors [23,24]. The principal targets of PEDV infection are intestinal epithelial cells [25], which are the major site of dietary iron absorption [26]. Whether piglets’ iron metabolism influences PEDV infection and/or their susceptibility to PEDV remain largely unknown.

In this study, we investigated the relationship between iron deficiency and PEDV infection *in vivo* and *in vitro* using newborn piglets and established cell lines. We found that iron levels influence the process of PEDV infection by affecting the expression of TfR1, and that higher expression of TfR1 in the intestinal epithelial cells (due to iron deficiency) contributes to the susceptibility of newborn piglets to PEDV.

## Results

### Distribution of PEDV and TfR1 in the small intestines of infected newborn piglets

**Fig 1:**
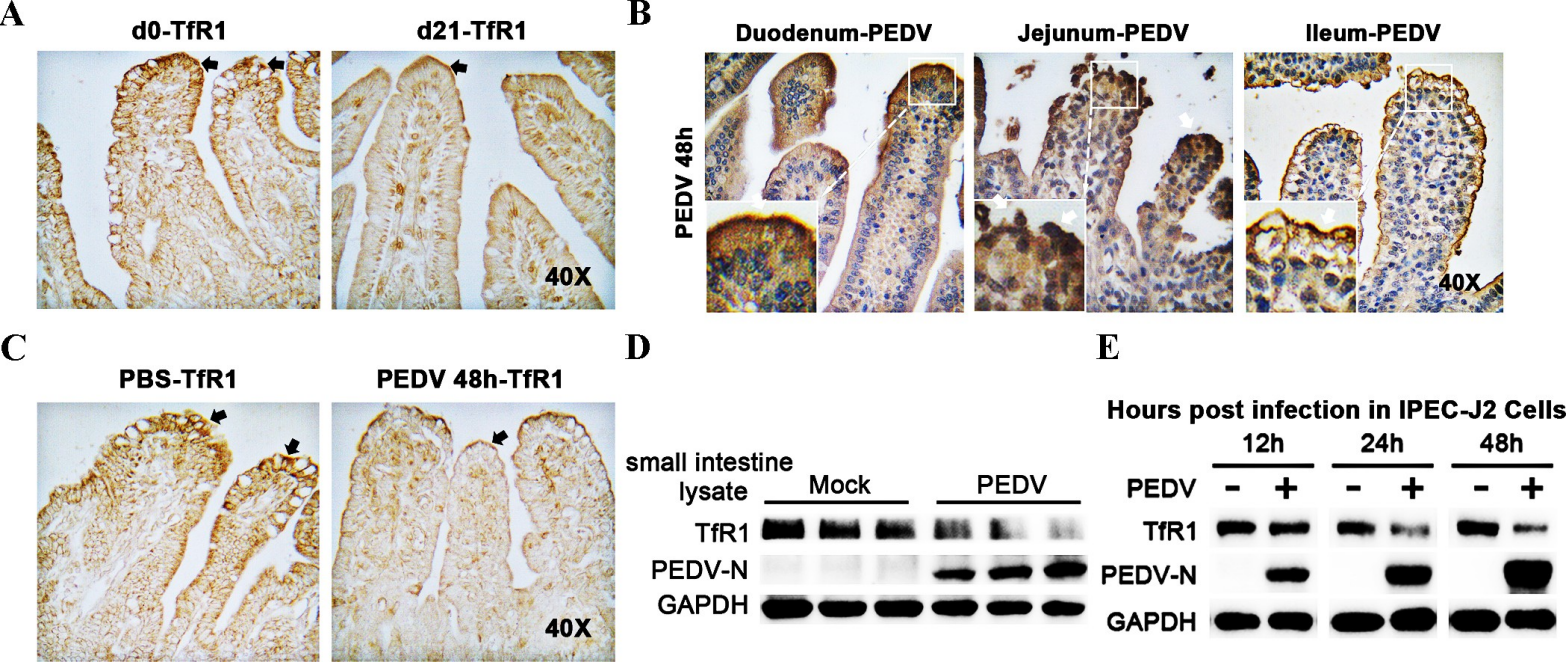
Distribution of PEDV and TfR1 in the small intestine of infected piglets Tissues were fixed with Bonn's liquid, embedded in paraffin, and sectioned into 4 μm sections. Immunohistochemistry was performed on the paraffin sections, which were then sealed with a cover slip. (A) Distribution of TfR1 in the small intestine of the day (d) 0 and d21 pigs (Magnification × 40). TfR1 stained deep yellow-brown (black arrows) is located high in the apical region of the intestines (B) Distribution of PEDV in the small intestine of newborn piglets (Magnification, × 40). PEDV-N protein stained deep yellow-brown (white arrows). (C) TfR1 staining (black arrows) in the uninfected control (PBS) group versus TfR1 staining in the PEDV infected group (Magnification, × 40). (D) Western blots of TfR1 and PEDV-N protein expression in the small intestine of the PBS control group and PEDV infected group. (E) IPEC-J2 cells were uninfected or infected with PEDV and harvested at 12, 24, and 48 h p.i. The cell lysates were analyzed by western blotting using anti-TfR1, anti-PEDV-N, and anti-GAPDH antibodies as probes.

Because they are born with low iron reserves, newborn piglets commonly suffer from iron deficiency. Perls’ Prussian blue staining demonstrates that the iron levels (blue granules) in the intestinal epithelial cells of d0 pigs were far less than in d31 pigs (S1 Fig). We found a greater distribution of transferrin receptor 1 (TfR1) in the apical surface of the villus in the epithelial cells in d0 piglets compared with d21 (Fig 1A) and d31 old pigs (S2 Fig). As a key molecule in the absorption of iron, the higher expression of TfR1, caused by the iron deficiency may account for the susceptibility of newborn piglets to PEDV.

Newborn piglets were assigned to either the mock infected control group or the PEDV infected group; piglets were infected by oral inoculation. Clinical signs of infection, including watery diarrhea and vomiting, were observed in the PEDV piglets at 48 hours post infection (h p.i.). All piglets were then anesthetized and sacrificed. As PEDV infects the epithelial cells of the small intestine in newborn piglets [25], we observed sections of the duodenum, jejunum, and ilium by immunohistochemistry; anti-PEDV-N antibody was used to identify infection. The virus was distributed mainly in the villi of the jejunum, but around the epithelial cells of duodenum, jejunum, and ileum as well (Fig 1B). No PEDV-N antigen-positive cells were detected in tissues of negative control piglets using anti-PEDV-N antibody, and no PEDV-N antigen-positive cells were detected PEDV infected piglets’ jejunum section using anti-mouse IgG as the primary antibody (S3 Fig). Immunohistochemistry was used to determine whether the expression of TfR1 in the apical surface of the villi is altered by PEDV infection. Results showed that expression of TfR1 was decreased in the PEDV infected piglets over the control piglets (Fig 1C). Western blotting of intestinal lysates was used to confirm the reduction in TfR1 in PEDV infected piglets (Fig 1D). The relationship between TfR1 and PEDV infection was examined *in vitro* using IPEC-J2 and Vero cells. Results showed that in both cell lines TfR1 levels were reduced as PEDV infection proceeded (Fig 1E) and (S4 Fig).

### TfR1 co-localizes with PEDV during infection

**Fig 2:**
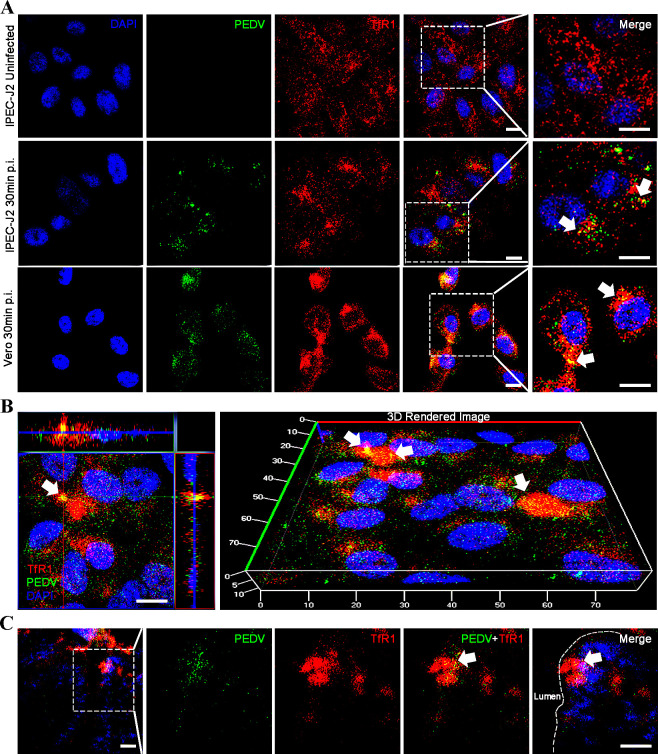
PEDV co-localizes with TfR1. (A) IPEC-J2 and Vero cells were infected with PEDV and fixed at 30 min p.i. Cells were then stained for confocal microscopy using rabbit anti-TfR1 Ab, followed by Dylight 649-conjugated goat anti-rabbit IgG (red) and FITC-conjugated anti-PEDV polyclonal antibody (green). The nuclei were stained with DAPI (blue). The white arrows indicate co-localized signals (scale bar = 10 μm). (B) IPEC-J2 cells were infected with PEDV and cultured for 1 h. The cells were then stained for confocal microscopy using rabbit anti-TfR1 pAb and mouse anti-PEDV N mAb, followed by Dylight 649-conjugated goat anti-rabbit IgG (red) and Dylight 488-conjugated goat anti-mouse IgG (green). Nuclei were stained with DAPI (blue). The panel shows a three-dimensional rendering of a representative field obtained using Imaris 7.2 software; the white arrows indicate co-localized signals (scale bar = 10 μm). (C) Jejunum segments of anesthetized newborn piglets were injected with PEDV (0.2 ml/segment). Indirect immunofluorescence images of PEDV and TfR1 in the infected segments at 1 h p.i. Fixed frozen sections were stained for confocal microscopy using rabbit anti-TfR1 Ab, followed by Dylight 649-conjugated goat anti-rabbit IgG (red) and FITC-conjugated anti-PEDV polyclonal antibody (green). Nuclei were stained with DAPI (blue). The white arrows indicate co-localized signals (Scale bar = 20 μm).

We used confocal microscopy to visualize the subcellular locations of PEDV and TfR1 at the early stages of infection (30 min and 1 hour post infection). PEDV was directly visualized using FITC-PEDV, and Dylight 488-PEDV for infection, TfR1 was indirectly visualized with the 649-conjugated anti-TfR1 antibody. Results showed that at 30 m p.i. TfR1 co-localized with PEDV in IPEC-J2 and Vero cells (Fig 2A). At 1 h p.i. TfR1 appeared to re-localize, cluster, and internalize in IPEC-J2 cells (Fig 2B). Importantly, we found that PEDV co-localized with TfR1 in the jejunum segments taken from infected piglets in the ligated loop experiments (Fig 2C).

### Altering the levels of intracellular labile iron affects TfR1 mediated PEDV infection

**Fig 3:**
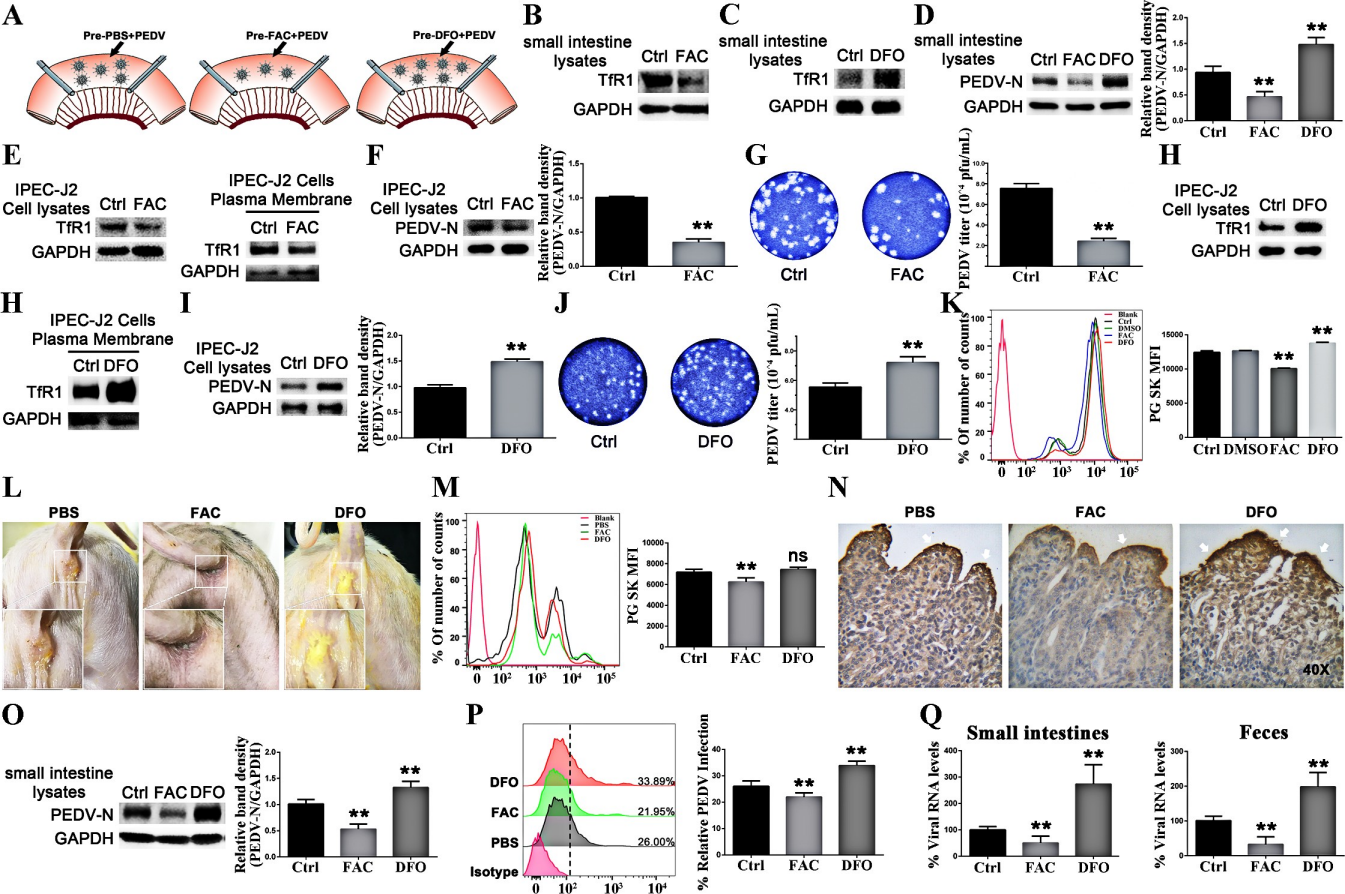
Levels of intracellular labile iron affect TfR1 mediated PEDV infection. (A) Anesthetized newborn piglets were subjected to intestinal ligation followed by treatment with PBS, FAC, DFO, PBS+PEDV, FAC+PEDV, and DFO+PEDV. Three hours after treatment with PBS, FAC, or DFO, the piglets’ jejuna were injected with PEDV, all piglets were sacrificed 6 h later. (B) Western blots of lysates of ligated segments treated with PBS or FAC demonstrate the reduced expression of TfR1 in FAC treated cells. (C) Western blot of the same experiments using DFO shows the increased expression of TfR1. (D) FAC reduced replication of PEDV-N protein and DFO increased replication of PEDV-N protein. (E) Western blotting shows a decrease in total and membrane TfR1 levels in IPEC-J2 cells treated with FAC. (F) Western blotting, at 24 h p.i of FAC treated IPEC-J2 cells infected with PEDV (MOI 1). Control is PBS treated/PEDV infected. (G) Culture supernatants from Figure G were titered by plaque assay in Vero cells. Plaque assays were developed 3 days after infection. (H Western blotting shows the increase in total and membrane TfR1 levels in IPEC-J2 cells treated with DFO. (I) Western blotting, at 24 h p.i of DFO treated IPEC-J2 cells infected with PEDV (MOI 1). Control is PBS treated/PEDV infected. (J) Culture supernatants from Figure K were titered by plaque assay in Vero cells. Plaque assays were developed 3 days after infection. (K) Flow cytometry of intracellular iron in IPEC-J2 cells treated with FAC and DFO. The fluorescence profile of each sample and the quantitative analysis demonstrate the increase and decrease in intracellular iron with FAC and DFO treatment respectively. (L) The arse of the piglets’ observation photos. (M) Flow cytometry of intracellular iron in primary porcine enterocytes from newborn piglets treated with FAC and DFO. (N) Distribution of PEDV in the jejunum of different groups’ newborn piglets (Magnification, × 40). PEDV-N protein stained deep yellow-brown (white arrows). (O) FAC reduced replication of PEDV-N protein and DFO increased replication of PEDV-N protein in the jejunum of newborn piglets. (P) Flow cytometry of primary porcine enterocytes from pretreated with FAC or DFO newborn piglets then challenged with PEDV. the replication of PEDV-N protein was detected by western blotting. (Q) Detection of RNA levels of PEDV in the jejunum of piglets and feces by qRT-PCR. The PEDV-N to GAPDH ratios were normalized to controls. The error bars represent standard deviations. * 0.01 < p < 0.05, ** p < 0.01 (compared to the Ctrl group).

We also found a significant reduction in intracellular iron levels at 24 h after PEDV infection with IPEC-J2 cells (S5 Fig). Building on the finding that PEDV infection influence iron levels via TfR1 mediated PEDV entry, we next investigated whether altering iron levels affected TfR1 mediated PEDV infection. As PEDV mainly infects the small intestine [27,28], we selected it as the site for further experiments. Fig 3A illustrates the *in vivo* treatments to the ligated small intestines of newborn piglets. Ferric ammonium citrate (FAC) has been shown to increase iron stores. Segments of small intestine were pretreated with FAC for 3 h, as expected, the intestinal absorption of iron increased and the expression of TfR1 decreased as shown by western blotting of intestinal lysates (Fig 3B). Deferoxamine (DFO) is an iron chelator and can be used to create an iron deficiency. Intestinal segments treated with DFO showed increased expression of TfR1 (Fig 3C). In parallel intestinal segments, FAC or DFO treated and untreated, were injected with PEDV for 6 h. PEDV-N protein expression was analyzed by western blotting and showed that PEDV infection was reduced in piglets treated with FAC, while intestinal segments treated with DFO were showed increased levels of PEDV infection (Fig 3D).

We carried out analogous experiments *in vitro* using IPEC-J2 cells. As can be seen in the western blots (Fig 3E) of cell lysates, total TfR1 and membrane TfR1 levels are reduced in FAC treated cells. PEDV infection levels are also reduced in FAC treated cells, as shown by western blotting (Fig 3F) and plaque assay (Fig 3G). As with the *in vivo* experiments, total and membrane TfR1 expression was increased in the DFO treated cells (Fig 3H), as was PEDV infection (Fig 3I and 3J). Changes in the intracellular iron content of IPEC-J2 cells treated with FAC and DFO were confirmed by flow cytometry. The overlay of the fluorescence signals shows that FAC treatment resulted in increased intracellular labile iron, as can be seen by the quenched Phen Green SK signal, and DFO treatment resulted in a decrease in the intracellular labile iron, can be seen by the increased PG SK signal (Fig 3K).

Next, we explored whether iron can protect newborn piglets from the PEDV challenge. Newborn piglets’ intramuscular injection at 2 hours after birth were challenged with PEDV at five hours after birth. Lethargy and diarrheic feces in PEDV-challenged DFO group piglets were first observed at 13 h p.i., and expressed clinically as severe watery diarrhea with vomiting thereafter by 16 h p.i. In contrast, mild diarrhea and lethargy could be seen in the PEDV-challenged PBS group piglets. Strikingly, in the PEDV-challenged FAC treatment group piglets, there were no obvious clinical signs (Fig 3L). All piglets were sacrificed when severe watery diarrhea was observed in the PBS group at 24 h p.i. Flow cytometry also showed that changes in the intracellular iron content of jejunal enterocytes from piglets were significantly increased after intramuscular injection with FAC, while the overlay of the fluorescence signals shows that DFO treatment resulted in a no significant decrease in the intracellular labile iron (Fig 3M). PEDV antigen was detected by immunohistochemical staining in the jejunal epithelial cells of challenged newborn piglets. Intense antigen labeling was apparent in PEDV-challenged DFO group piglets, while mild detection was observed in the jejunum of the PEDV-challenged PBS group piglets, and diminished immunolabeling was visible in PEDV-challenged FAC group piglets (Fig 3N). Western blot results further validated the PEDV level in the jejunum of different groups piglets, and a significant quantity of PEDV N protein was detected in PEDV-challenged DFO group piglets, and the intensity of PEDV-challenged PBS group piglets was signally diminished (Fig 3O). Besides, flow cytometry demonstrates that the primary porcine enterocytes of PEDV-challenged FAC group piglets reduce PEDV infection and DFO treatment results in a marked PEDV infection (Fig 3P). Finally, the level of viral RNA expression in jejunum and feces were quantified by quantitative reverse transcription-polymerase chain reaction (qRT-PCR). PEDV RNA expression in the jejunum and feces of PEDV-challenged DFO group piglets was significantly higher than that in the other groups, and low-level RNA expression was detected in PEDV-challenged PBS group piglets (Fig 3Q). Overall, the results of this experiment suggested that passively supplement iron contributed to the protection of neonatal piglets against PEDV infection.

### Inhibiting TfR1 expression decreases infection by PEDV

**Fig 4:**
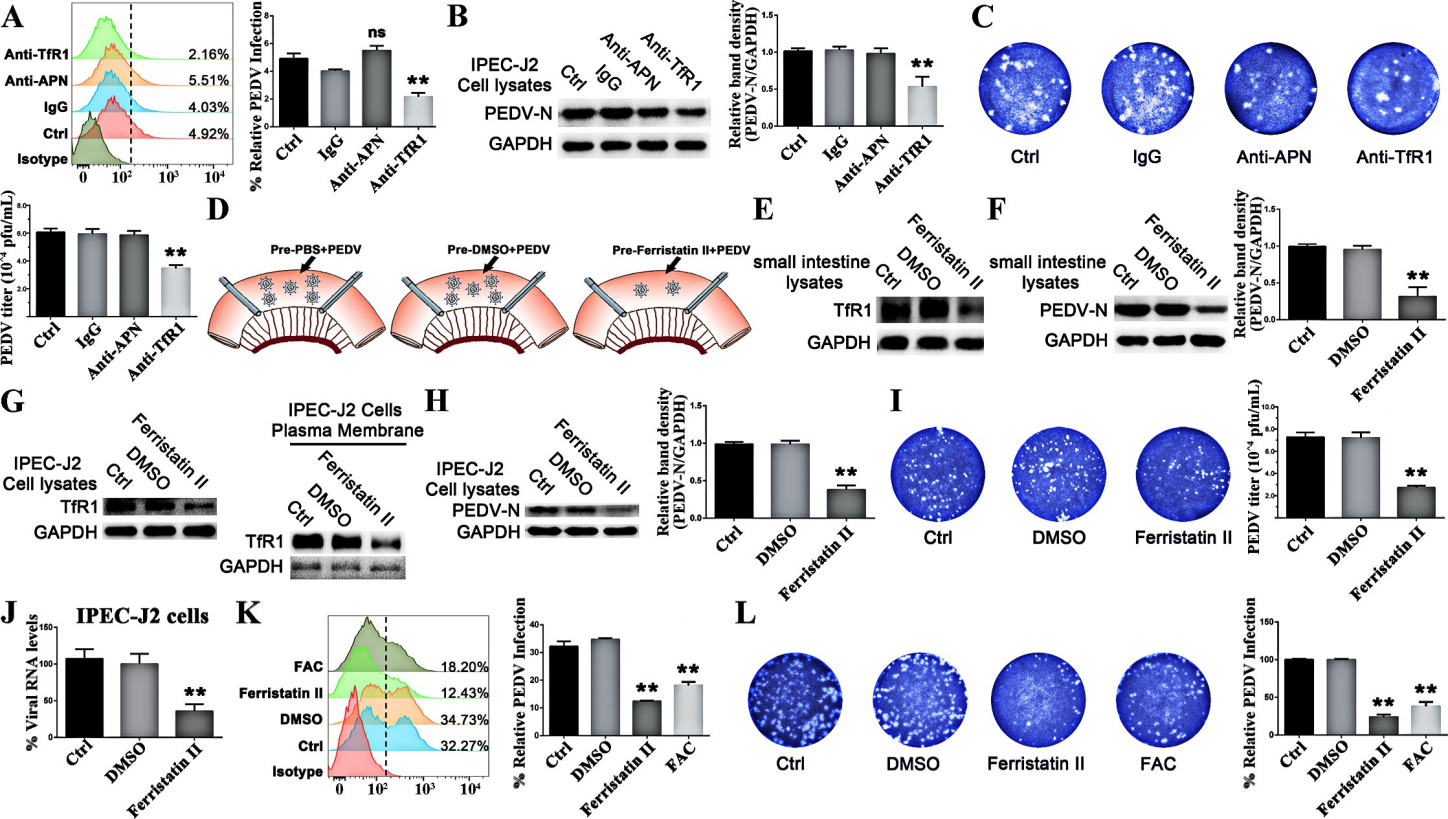
Inhibiting TfR1 protein inhibits PEDV Infection. (A and B) IPEC-J2 cells were pre-incubated with anti-TfR1 or anti-pAPN Ab for 1 h at 37°C. The entry of PEDV (at 1 h p.i. MOI 1) was quantitated by flow cytometry, and the replication of PEDV-N protein was detected by western blotting at 24 h p.i. (C) The virus in the culture supernatants from Figure B was titered by plaque assay in Vero cells. (D) Schematic of the ligated loop experiments in anesthetized newborn piglets. Jejunum segments were treated with PBS, DMSO, or ferristatin II for 3 h then challenged with PEDV for another 6 h. (E and F) Western blots of TfR1 expression from ferristatin II treated ligated segments, and the replication of PEDV-N protein. (G) Western blots showing total and membrane TfR1 levels in IPEC-J2 cells treated with ferristatin II. (H) Western blot at 24 h p.i. of IPEC-J2 cells pretreated with ferristatin II for 3 h then infected with PEDV (MOI 1). (I) The virus in the culture supernatants from Figure I was titered by plaque assay in Vero cells. (J) Detection of RNA levels of PEDV at 24 h p.i. in the of IPEC-J2 cells pretreated with ferristatin II then infected with PEDV by qRT-PCR. (K) Flow cytometry at 24 h p.i. of IPEC-J2 cells pretreated with ferristatin II or FAC then infected with PEDV. (L) Plague assays of Vero cells treated with ferristatin II or FAC then infected with PEDV. The PEDV-N to GAPDH ratios were normalized to controls. The data shown are means ± SD from three independent experiments. (* 0.01 < p < 0.05, ** p < 0.01).

To confirm that changes in TfR1 expression impact PEDV infection, we blocked and inhibited its expression. IPEC-J2 cells were treated with an anti-TfR1 antibody or an anti-pAPN antibody (porcine aminopeptidase N, a PEDV receptor) before incubation with PEDV. At 1 h p.i. flow cytometry results showed that only blocking TfR1 resulted in decreased PEDV entry while blocking pAPN had no obvious effect (Fig 4A). Western blot and plaque assay results demonstrated that there was no difference in outcome at 24 h p.i. (Fig 4B and 4C). There was no observable cytopathic effect in group Mock (without PEDV infection) and group anti-TfR1 except for group Ctrl, group IgG, and group anti-pAPN at 24 h PEDV infection in Vero cells (S6 Fig). Ferristatin II, which causes TfR1 degradation [29], was used to test the role of TfR1 in PEDV infection. Zhang *et al*. demonstrated that a 50 uM dose did not result in detectable cytotoxicity [30]. We found that in ligation loop experiments and *in vitro* with IPEC-J2 cells, TfR1 expression was significantly reduced with ferristatin II treatment (Fig 4E and 4G). Western blotting (Fig 4F and 4H) and plaque (Fig 4I) results also showed that PEDV infection was significantly decreased whether total TfR1 or membrane TfR1 (Fig 4G) was degraded. qRT-PCR also showed PEDV RNA expression was significantly decreased with ferristatin II treatment (Fig 4J). Flow cytometry also showed that PEDV infection in IPEC-J2 cells was significantly decreased after pretreating with ferristatin II or FAC (Fig 4K). Plaque assays using Vero cells pretreated ferristatin II or FAC then infected with the same countable PEDV, also showed that PEDV infection was reduced in treated cells over untreated and DMSO treated cells (Fig 4L).

**Fig 5:**
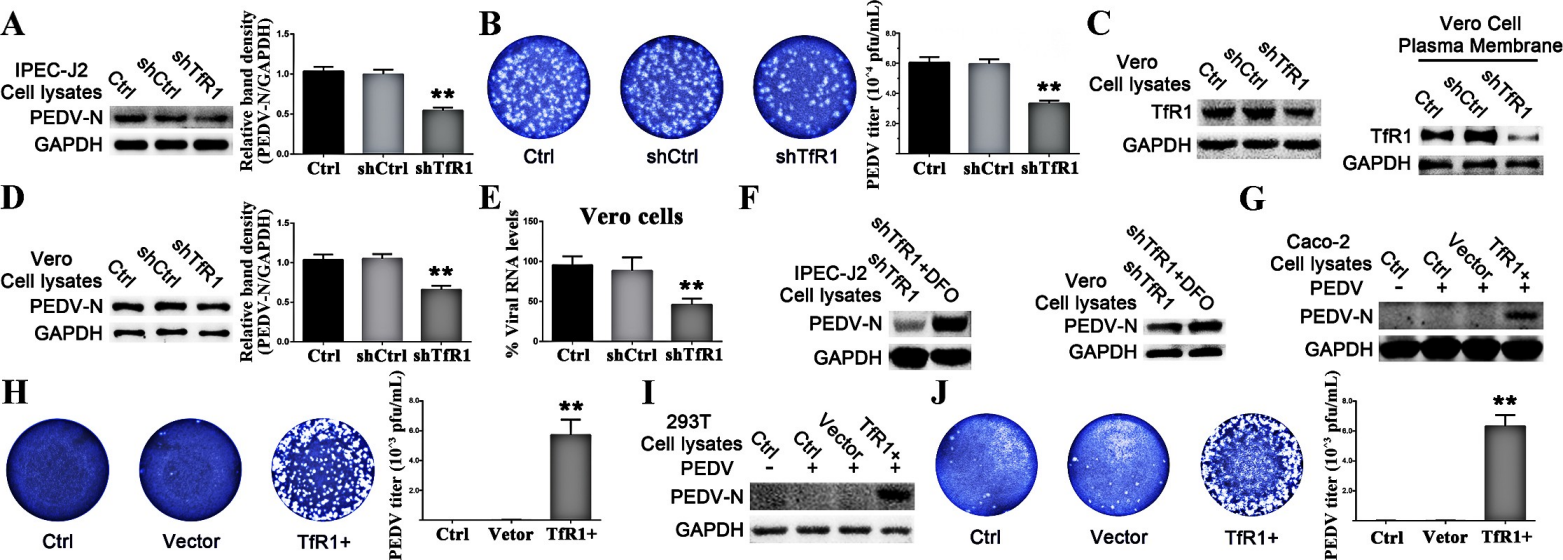
Increased TfR1 expression enhances infection by PEDV. (A) Western blot at 24 h p.i. of TfR1 silenced IPEC-J2 cells infected with PEDV. (B) The virus in culture supernatants from Figure A was titered by plaque assay in Vero cells. (C) Western blot of pLVX-shRNA-TfR1 transfected Vero cells demonstrating the efficiency of TfR1 silencing. (D) Western blot at 24 h p.i. of stably TfR1-silenced Vero cells infected with PEDV. (E) TfR1-silenced Vero cells were infected with PEDV (MOI 1) at 24 h, pLVX-shRNA-Ctrl and normal cells served as controls. PEDV-N mRNA levels were quantified by RT-PCR. (F) Western blot at 24 h p.i. of TfR1-silenced IPEC-J2 cells and Vero cells treated with DFO and then infected with PEDV. Respectively, western blot and plaque assay at 24 h p.i. of TfR1-expressing Caco-2 cells (G and H) and HEK 293T cells (I and J) infected with PEDV. The PEDV-N to GAPDH ratios were normalized to controls. The data shown are means ± SD from three independent experiments. (* 0.01 < p < 0.05, ** p < 0.01).

shRNA specifically targeting TfR1 was used to inhibit TfR1 expression in IPEC-J2 cells [30] and Vero cells. Western blotting showed both total TfR1 and membrane TfR1 levels were effectively reduced compared to cells transfected scrambled shRNA and untransfected Vero cells (Fig 5C). PEDV infection was decreased in IPEC-J2 cells after TfR1 knocking down, as shown by western blot and plaque assay (Fig 5A and 5B). Similar results were observed by western blot and qRT-PCR in Vero cells (Fig 5D and 5E). These results demonstrate that TfR1 is involved in PEDV entry.

### Increased TfR1 levels enhance PEDV infection

Because DFO treatment prompts increased expression of TfR1, we posited that DFO treatment of TfR1 silenced cells would restore the cells' susceptibility to PEDV infection. Fig 5F demonstrates that the DFO treatment of TfR1 silenced cells does result in higher PEDV infection levels in IPEC-J2 and Vero cells. Given these results, we speculated that higher levels of TfR1 may promote infection in cells normally refractory to PEDV, hence, Caco-2 (Fig 5G and 5H) and HEK 293T (Fig 5I and 5J) cell lines were used. We found that in both cell lines, overexpression of TfR1 resulted in PEDV infection.

### PEDV interacts with endogenous TfR1

**Fig 6:**
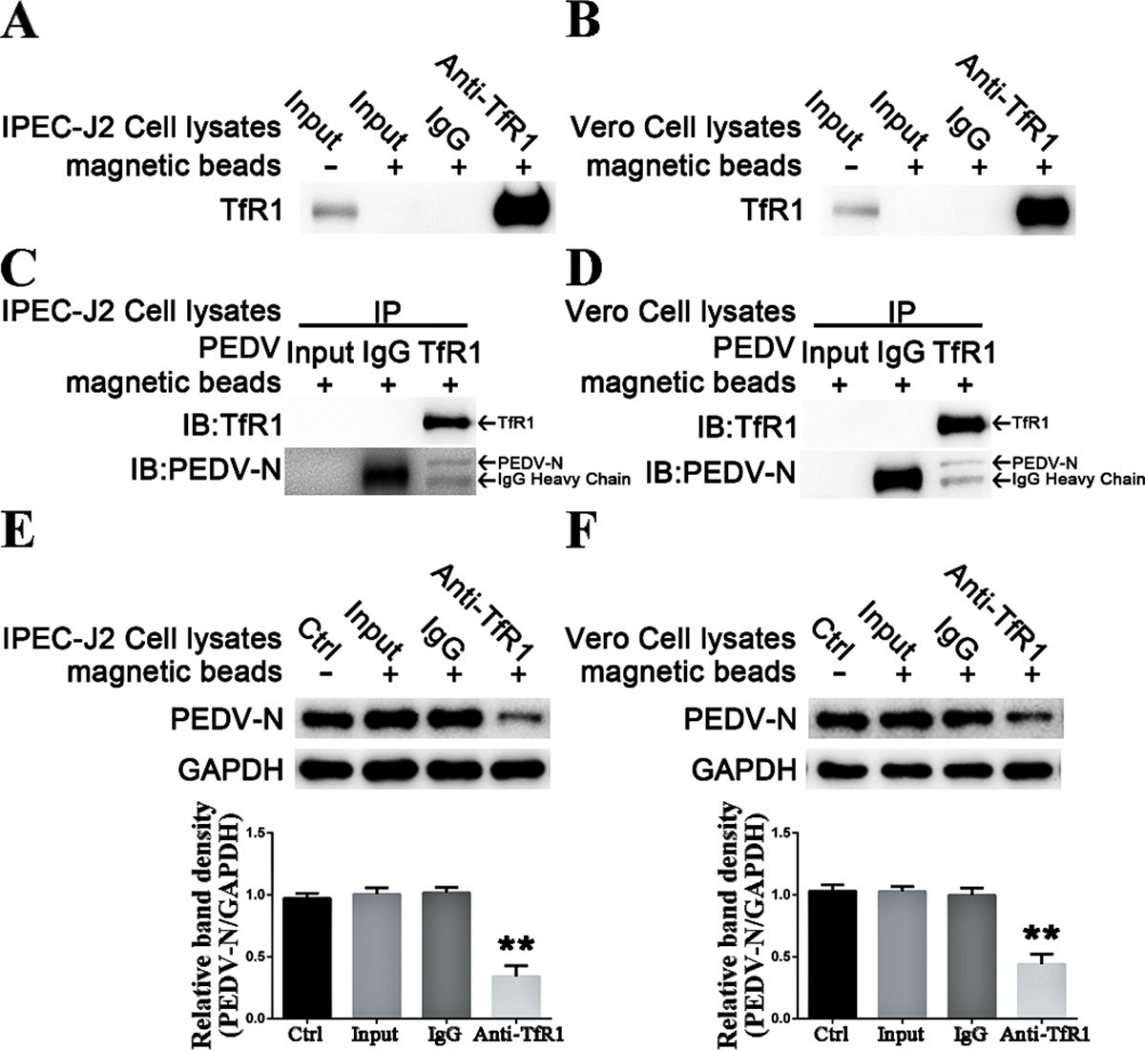
PEDV interacts with endogenous TfR1. (A and B) Western blots of the endogenous TfR1 from IPEC-J2 or Vero cell lysates bound to anti-TfR1 coated magnetic beads. (C and D) Western blots N protein precipitated by the TfR1 coated beads. (E and F) Western blots at 24 h p.i. of cells infected with PEDV (MOI 1) that had been pre-incubated with the TfR1 coated beads. The PEDV-N to GAPDH ratios were normalized to control conditions. Data shown are means ± SD from three independent experiments. (* 0.01 < p < 0.05, ** p < 0.01).

To investigate the hypothesis that PEDV directly interacts with endogenous TfR1, which is then used to assist PEDV entry into intestinal epithelial cells, magnetic beads bound to anti-TfR1 antibody were used to capture TfR1 from IPEC-J2 and Vero cells; empty magnetic beads (without antibody) and magnetic beads bound to allogeneic anti-rabbit IgG were used as negative controls (Fig 6A and 6B). Only beads with captured TfR1 bound PEDV (Fig 6C and 6D). Pre-incubating PEDV (MOI 5) with the precipitated endogenous TfR1, blocked viral replication as shown by western blotting and plague assays done at 24 h p.i. (Fig 6E and 6F). These data indicated that endogenous TfR1 interacts with PEDV.

### Extracellular TfR1 interacts with PEDV S1 protein *in vitro*

**Fig 7:**
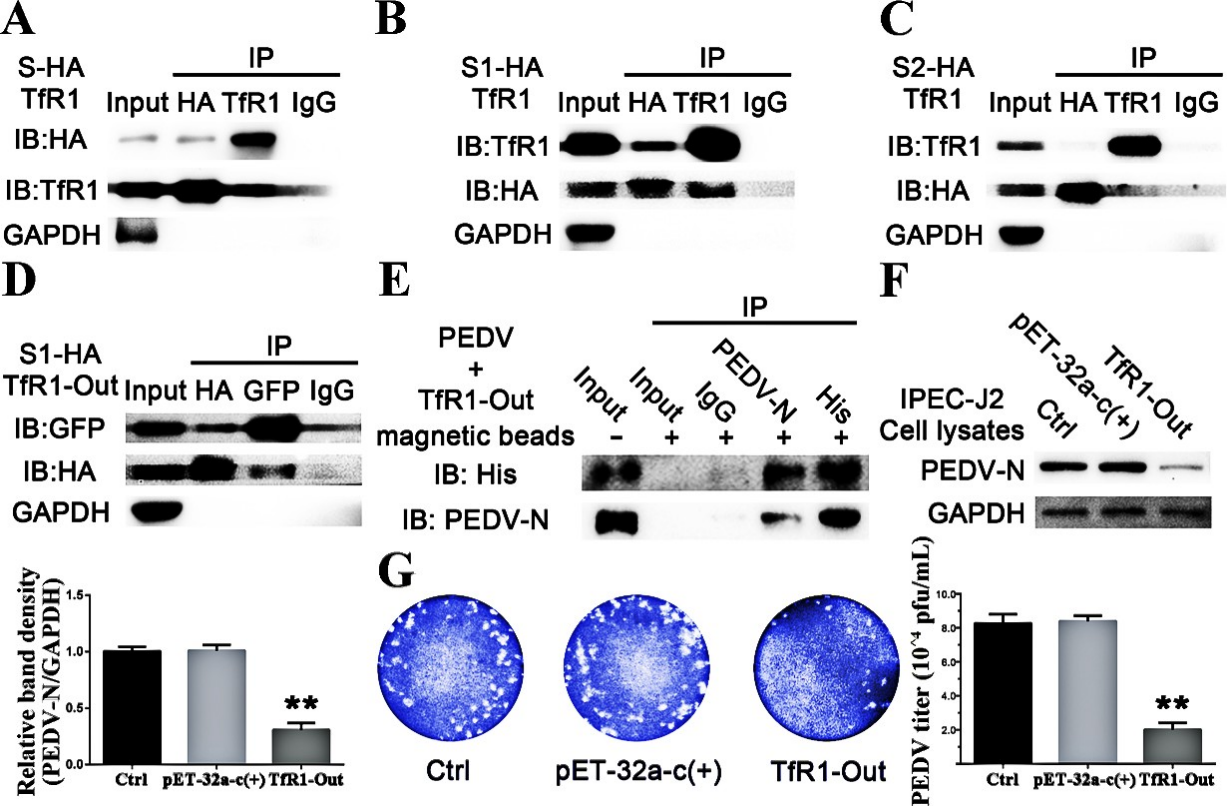
Extracellular TfR1 interacts with PEDV S1 protein in vitro. (A) Western blot of co-immunoprecipitations from lysates of 293T cells co-transfected with plasmids expressing TfR1 and PEDV S-HA. (B and C) Western blots of co-immunoprecipitations from lysates of 293T cells co-transfected with plasmids expressing TfR1and PEDV S1-HA or PEDV S2-HA. (D) Western blot of co-immunoprecipitations from lysates of 293T cells co-transfected with plasmids expressing PEDV S1-HA and GFP-TfR1-Out. (E) Western blot of the immunoprecipitation of purified His-TfR1-Out protein incubated with PEDV. His-TfR1-Out was precipitated with anti-PEDV-N coated beads, and PEDV N protein was precipitated with anti-His coated beads. (F and G) Respectively, western blot and plaque assays at 24 h p.i. of TfR1-Out treated IPEC-J2 cells subsequently infected with PEDV. The ratio of PEDV-N to GAPDH was normalized to controls. Data shown are means ± SD from three independent experiments. (* 0.01 < p < 0.05, ** p <0.01).

The spike (S) protein of PEDV is a type I transmembrane glycoprotein, it consists of two domains, S1 and S2 which are responsible for binding and fusion respectively [24,31,32]. To investigate the interaction between the S protein and TfR1 we constructed plasmids encoding the full length S protein, the S1, and S2 domains, and TfR1. Co-immunoprecipitations (Co-IPs) were performed from lysates of HEK 293T cells co-transfected with plasmids expressing PEDV S-HA and TfR1, PEDV S1-HA and TfR1, and PEDV S2-HA and TfR1 respectively. The results confirmed that full length S and truncated S1 interact with TfR1 (Fig 7A and 7B), and there is no interaction between S2 and TfR1 (Fig 7C). To explore more closely the interaction of S1 and TfR1, we constructed a plasmid encoding only the extracellular region of TfR1 (TfR1-Out). Co-IPs were done on lysates of cells co-transfected with PEDV S1-HA and TfR1-Out, and showed that the extracellular region of TfR1 interacts with PEDV S1 *in vitro* (Fig 7D). Lastly, 200 ng/ml of purified His-tagged TfR1-Out fusion protein, which had been expressed in *E*. *coli*, was pre-incubated with PEDV for 2 h at 37°C. Western blots show that PEDV combined with the TfR1-Out protein (Fig 7E), western blotting and plaque assays show that PEDV replication was inhibited (Fig 7F and 7G).

### TfR1 internalization is mediated by Src and cellular cholesterol during PEDV infection

**Fig 8:**
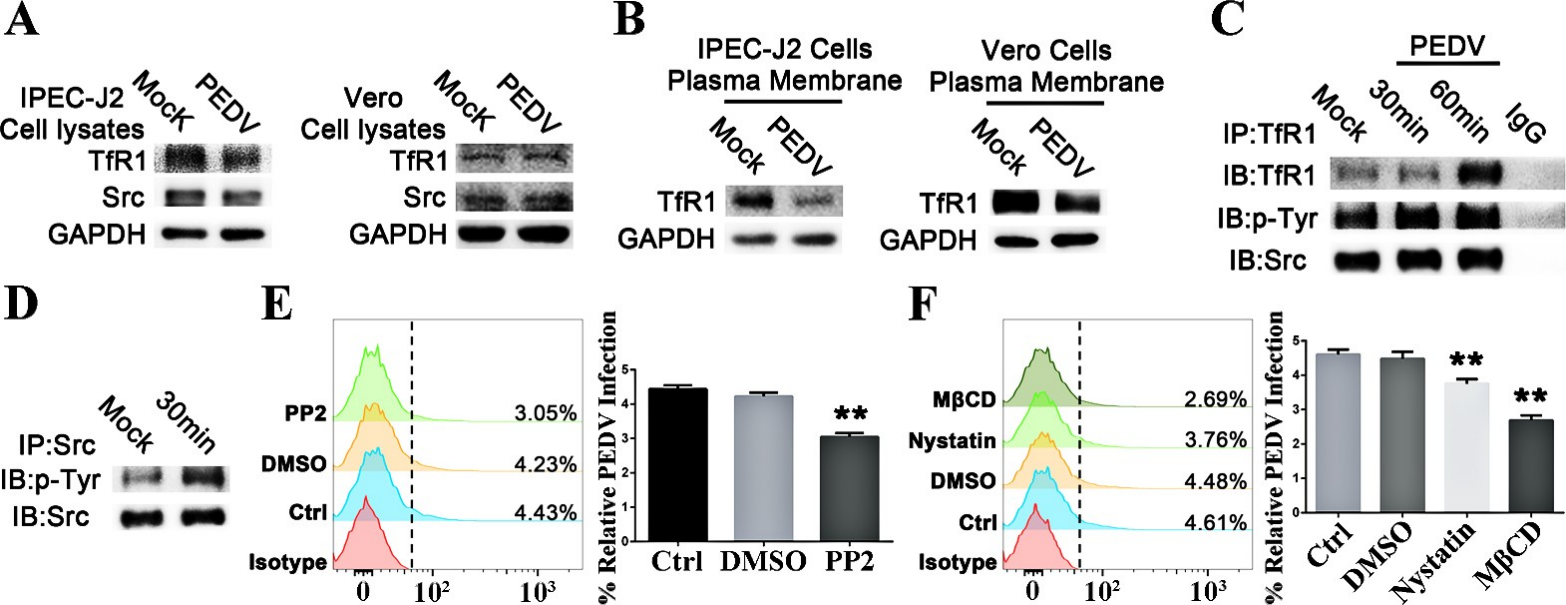
TfR1 internalization is associated with Src and cellular cholesterol during PEDV infection. (A) Western blots of membrane TfR1 levels in PEDV infected IPEC-J2 and Vero cells respectively. (B) Western blots of TfR1 and Src in infected IPEC-J2 and Vero cells respectively. (C) Western blot of the immunoprecipitation of TfR1, p-Tyr, and Src from PEDV infected IPEC-J2 cells. (D) Western blot of the immunoprecipitation of p-Tyr and Src from PEDV infected IPEC-J2 cells. (E) Flow cytometry of internalized PEDV in IPEC-J2 cells pretreated with the Src inhibitor PP2 then subsequently infected. (F) Flow cytometry of internalized PEDV in IPEC-J2 cells pretreated with nystatin or MβCD then subsequently infected with PEDV.

The internalization of TfR1 is regulated by Src kinase [33,34]. The expression of total TfR1 and Src in IPEC-J2 cells and Vero cells remained unchanged during the 1 hour of PEDV infection (Fig 8A). To further explore the role of TfR1 in PEDV entry, we extracted the membrane domain of TfR1 from infected IPEC-J2 and Vero cells at 1 h p.i., and found that the levels membrane TfR1 was decreased (Fig 8B), suggesting that TfR1 endocytosis was activated after S1 binding to TfR1. Lysates of IPEC-J2 cells infected with PEDV for 30 or 60 minutes were immunoprecipitated with anti-TfR1 or anti-Src antibody followed by immunoblotting for TfR1 or Src respectively. The results (Fig 8C) show that Src can bind to TfR1, and that PEDV infection resulted in an increased interaction of TfR1 with Src. TfR1 is tyrosine phosphorylated in IPEC-J2 cells and Fig 8C and 8D confirm that PEDV infection increased TfR1 tyrosine phosphorylation (p-Tyr). These results reveal that Src and TfR1 are constitutively bound after PEDV infection. To test whether Src is essential for the process of TfR1 mediated PEDV entry, IPEC-J2 cells were treated with the Src inhibitor PP2 before PEDV infection. Flow cytometry shows that inhibiting Src greatly reduces TfR1-mediated PEDV infection (Fig 8E).

Clathrin-mediated endocytosis also plays a role in TfR1 internalization. Membrane cholesterol plays a critical role in clathrin-coated pit internalization, and acute cholesterol depletion causes a decrease in clathrin-mediated endocytic vesicles [35,36]. To examine the role of cholesterol in clathrin-mediated TfR1 internalization during PEDV infection, we studied the effect of acute cholesterol depletion, using methyl-β-cyclodextrin (MβCD) and nystatin, on the endocytic behavior of the TfR1. Flow cytometry demonstrates that acute cholesterol depletion results in a marked reduction in PEDV infection (Fig 8F). Importantly, inhibition of cholesterol by MβCD or nystatin abrogates the clustering of TfR1 caused by PEDV infection (S7 Fig).

## Discussion

The disease outcome and mortality rate from PEDV infection are inversely associated with the age of the pig [37]. PED epizootic has been characterized by high mortality rates among suckling piglets, but milder disease in older weaned and adult pigs [19,37–42]. However, the concrete mechanism that newborn piglets are particularly susceptible to PEDV infection with greater disease severity and deaths versus weaned pigs have not been clearly, the reasons for this are not well elucidated. Therefore, finding the age-related mechanism of PEDV entry would provide insight for the development of anti-PEDV therapeutics. PEDV is highly enteropathogenic and acutely infect villous epithelial cells of atrophied villi of small (the jejunum and ileum are the primary sites of infection) and large intestines [39,40,43], resulting in severe villous atrophy and malabsorptive diarrhea [44]. The abundance of innate and adaptive immune cells that reside in the mammalian gastrointestinal tract requires barrier and regulatory mechanisms that conserve tissue homeostasis [45]. Intestinal epithelial cells (IECs) are crucial regulators of barrier function and immune homeostasis. IECs not only create a physical barrier in host tissues but also integrate and transmit signals to locally regulate immune response at the intestinal barrier and promote the maintenance of intestinal homeostasis [46–48]. Intestinal function and barrier integrity are impaired during PEDV infection, potentially leading to secondary bacterial infections [49,50]. The apparent rate of regeneration of intestinal villus epithelium in 3-week-old pigs (2–4 days) is less than in newborn pigs (7–10 days), possibly causing a slower turnover of enterocytes in neonatal piglets (5–7 days) compared with that (2–3 days) in weaned pigs [51–53]. Virus production (the intestine of newborn piglets contained 10^2^−10^5^ times more virus than 3-week-old pigs) in the comparatively old cells of newborn piglets was greater than in the comparatively young cells of 3-week-old pigs. The delayed increase of intestinal stem cell numbers and proliferation of crypt cells in neonatal piglets (3 days post-PEDV infection) than in weaned pigs (1 day post-PEDV infection) and villous atrophy of 3-week-old and adult piglets were generally less than in newborn piglets [53]. The delayed recovery intestinal function may be involved in the susceptibility of newborn piglets to severe disease by PEDV infection, while nursery pigs infected with PEDV can recover digestive function within 7 days [49]. These relationships (among the kinetics of epithelial replacement, virus production and regeneration of villi) explain why severe diarrhea is more frequent and prolonged in newborn piglets than in older pigs. Besides, viral infections induce both innate and adaptive immunity, while the newborn mainly depends on innate immune responses to clear or reduce viral infection in the absence of previous exposure to pathogens, and therefore no adaptive immune responses [54]. The high susceptibility of neonates to severe disease from infections is deficient innate and adaptive immune responses [42,55,56]. In instances when PEDV infected deficiency in innate immunity in suckling pigs compared with weaned pigs, severe disease severity coincides with reduced innate immune responses results [57].

The newborn piglets are at risk for iron deficiency because their rapid rate of growth makes it difficult to store iron. Iron is an element necessary for almost all living organisms as it participates in a wide variety of metabolic processes, including oxygen transport (hemoglobin and myoglobin), the formation of heme enzymes, deoxyribonucleic acid (DNA) synthesis, and electron transport [58]. Iron deficiency has implicated as a factor associated with viral infections [5]. Our study aimed to investigate whether iron deficiency plays a role in the susceptibility of neonatal piglets to PEDV. In older pigs, TfR1 is normally found expressed in the epithelial cytoplasm of crypt epithelial cells [10,59,60]. In newborn piglets with iron deficiency, we found that TfR1 was distributed widely along with the epithelial layer in the small intestine, especially in the apical portion of the surface epithelium (Fig 1A). Therefore, we hypothesized that iron deficiency leads to increased expression of TfR1 in the intestine, which in turn increases the available targets for TfR1 mediated PEDV entry. This would account for the age sensitivity of newborn piglets to PEDV. In this study, the impact of iron status on PEDV infection was explored using intestinal ligation experiments and challenge protection test in newborn piglets (Fig 3). We found that newborn piglets infected with PEDV decreased when treated FAC (which increases iron levels) and increased when treated with DFO (an iron chelator) (Fig 3D and 3O). In vitro results were identical (Fig 3E–3J). We demonstrated that newborn piglets suffering from iron deficiency had high expression of TfR1 in the apical tissue of intestinal villi, which may directly contribute to their susceptibility to PEDV.

Virus entry is the first step of viral infection [61] and PEDV invades the intestinal epithelial cells via a receptor-mediated fusion mechanism. Previous studies reported that porcine aminopeptidase N (pAPN) and sialic acid have both been suggested as binding receptors for PEDV, which was reviewed by Li et al. [24]. Porcine aminopeptidase N (pAPN), distributed on enterocytes widely and probably on other tissues, irrespective of age and tissue [62], acts as a receptor for PEDV [24,63–65]. Li et al. showed that pAPN can bind PEDV and anti-pAPN antibodies can block PEDV infection [65]. Further research suggests that pAPN may not be the functional receptor, but rather it contributes to PEDV infection through its aminopeptidase activity [66–68]. This was further confirmed by the fact that PEDV infects pAPN-knockout pigs [69]. Besides, Sun et al., utilizing surface plasmon resonance (SPR) analyses showed that the functional pAPN ectodomain did not bind to either PEDV S1 nor S2 protein [70]. These divergent observations suggest that pAPN may be controversial as a genuine PEDV receptor and PEDV may use more than one receptor to infect host cells. Therefore, we hypothesis that TfR1 may be an entry receptor of PEDV.

In the results (Fig 4A–4C), PEDV infection was inhibited when TfR1 was blocked by pre-incubating with anti-TfR1 antibody, while there was no obvious effect on PEDV infection when pAPN was blocked. Furthermore, we found that PEDV infection induces TfR1 re-localization, clustering, and internalization (decreasing the membrane TfR1 levels) thereby mediating PEDV entry (Fig 2 and Fig 8A and 8B). As infection proceeded, total TfR1 expression decreased both *in vivo* and *in vitro* (Fig 1D and 1E). The surface spike (S) protein on coronaviruses mediates virus entry and determines virus tropism [71,72]. Like other coronaviruses, PEDV S protein is a type I glycoprotein composed of an S1 receptor binding unit and an S2 membrane fusion unit [66,73,74]. The S protein plays a pivotal role in regulating the interaction with specific receptors on host cells to mediate the virus entry [75,76]. In our study, we demonstrated the extracellular region of endogenous TfR1 interacts with the PEDV S1 protein but not S2 protein (Fig 7A–7D). We showed by pre-incubating PEDV with endogenous TfR1, that PEDV entry was blocked (Fig 6E and 6F). In the results (Fig 7F and 7G), preincubation of PEDV with TfR1-Out blocked only partially, but not completely, PEDV infection. Additionally, we also found that decreasing the expression of TfR1 with ferristatin II (Fig 4D–4J) or shRNA (Fig 5A–5E) impaired PEDV infection. What’s more, overexpression of TfR1 enhanced PEDV entry even in PEDV resistant Caco-2 and HEK 293T cells (Fig 5G–5J). Collectively, the data in this study suggest that TfR1 may be one of the receptors for PEDV infection.

Iron-bound Tf is internalized by TfR1 through clathrin-mediated internalization in clathrin-coated pits [77]. Membrane cholesterol also plays a critical role in clathrin-coated pit internalization [35]. Previous studies have shown that cholesterol depletion from the cellular membrane by methyl-β-cyclodextrin (MβCD) significantly impairs viral infection by affecting a post-adsorption step in the virus entry process that requires membrane rearrangement [78,79]. We found that the effects of acute cholesterol depletion by MβCD and nystatin, prevention of endocytic coated pit detachment and hampered membrane TfR1 rearrangement and clustering (Fig 8A–8D and S7 Fig), resulted in a marked reduction in the level of PEDV infection (Fig 8F). Src tyrosine kinase plays a crucial role in the coordination and facilitation of cell signaling pathways [80,81]; the endocytosis of TfR1 is a regulated process that requires activated Src kinase [34]. Consistent with the results of Jinlong Jian *et al*. [33], we found that TfR1 binds Src and showed that PEDV infection increased Src availability for TfR1 Tyr20 phosphorylation (Fig 8C and 8D). Interestingly, the Src inhibitor PP2 significantly reduced PEDV infection (Fig 8E).

**Fig 9:**
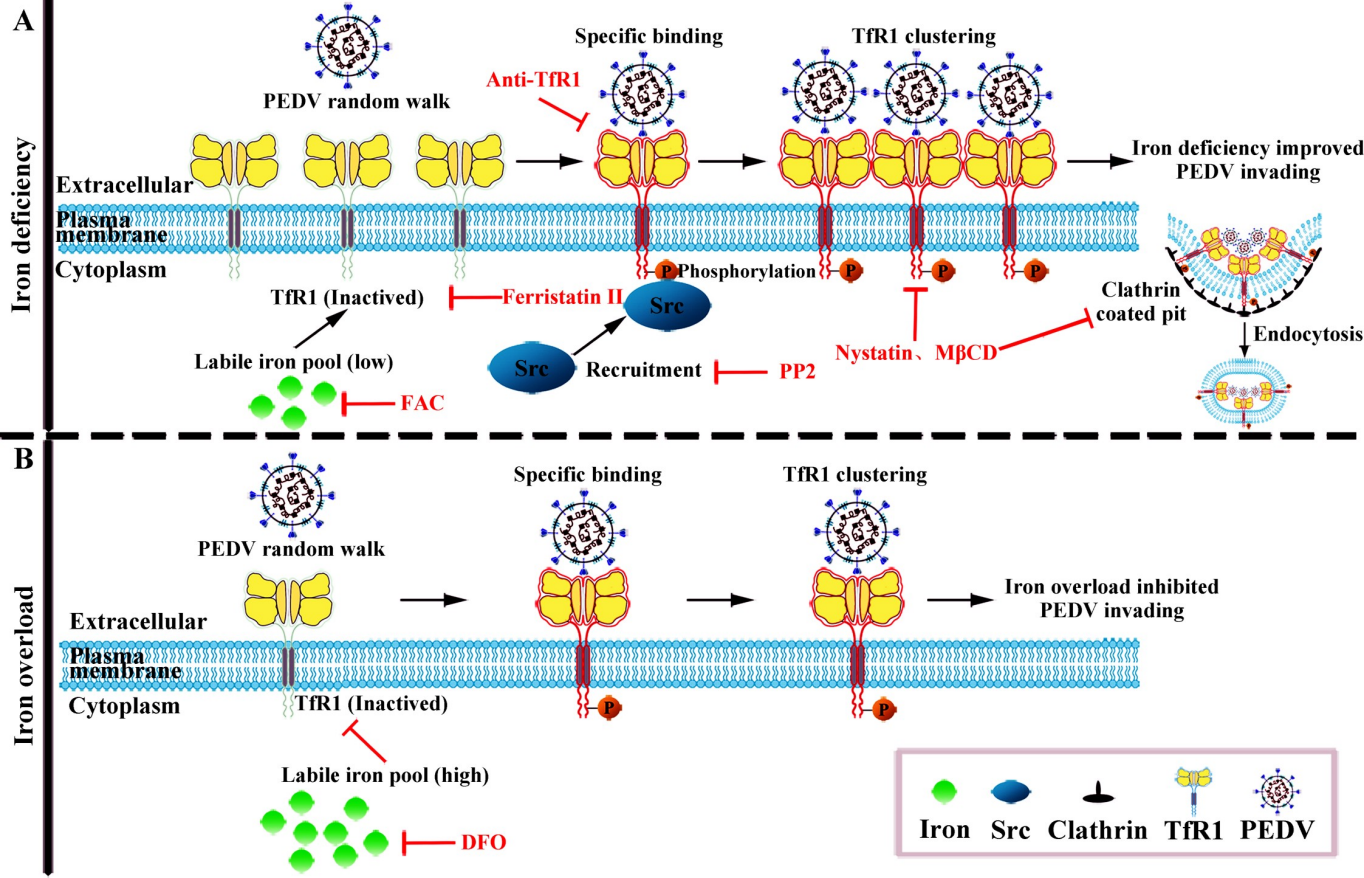
The schematic of intracellular iron levels influences the susceptibility of newborn piglets to TfR1 mediated PEDV infection. (A) Iron deficiency improved PEDV invading. High expression of TfR1 on the surface of intestinal epithelial cells in iron deficient newborn piglets, promotes PEDV infection. TfR1 may be a receptor for PEDV infection; the PEDV S1 protein interacts with the extracellular portion of TfR1, PEDV attachment causes TfR1 clustering, recruits Src kinase, actives TfR1 tyrosine 20 phosphorylation, and induces the formation of clathrin coated endocytic vesicles, which results in TfR1-bound PEDV internalization. (B) Iron overload inhibited PEDV infection. Under the conditions of iron overload, the expression of membrane TfR1 is decreased, thus curbing the PEDV invasion.

In summary, our results demonstrate that TfR1 may act as a PEDV receptor and that the levels of intracellular iron in intestinal epithelial cells may affect the expression of TfR1 and then indirectly affects PEDV infection (Fig 9). The high density of TfR1 levels on enterocytes may be a key factor contributing to the high level of PEDV susceptibility in newborn piglets with iron deficiency, and as newborns are commonly iron deficient, this also accounts for the age sensitivity of pigs to PEDV. The insights this work has provided will benefit the development of anti-PEDV therapies by iron supplementation for newborn piglets.

## Methods

### Ethics statement

All animal studies were approved by the Institutional Animal Care and Use Committee of Nanjing Agricultural University (SYXK-2017-0027), and followed the National Institutes of Health guidelines for the performance of animal experiments.

### Antibodies

Rabbit anti-TfR1 (Abcam). Mouse monoclonal anti-p-Tyr (PY20) (Santa Cruz Biotechnology). Rabbit anti-Src (Cell Signaling Technology). Rabbit anti-pAPN (ABclonal Biotechnology). Mouse monoclonal antibodies to His, HA, and GFP (CMCTAG, Milwaukee, USA). Mouse monoclonal anti-PEDV N protein was purchased from Medgene labs (FACS, 1:100; IF, 1:200; Western-blot, 1:1000). FITC-conjugated anti-PEDV polyclonal antibody was purchased from VMRD (1:200, PC-IFA-PEDV). Dylight 488 goat anti-mouse IgG (H+L) and Dylight 649 goat anti-rabbit IgG (H+L) were from MultiSciences (Lianke) Biotech, CO., LTD. Rabbit monoclonal anti-GAPDH and goat anti-rabbit IgG-HRP were from Bioworld Technology Inc (St. Louis Park, MN, USA). HRP-conjugated goat anti-mouse IgG (H+L) (Vazyme, Nanjing, China). Anti-rabbit IgG antibody (Beyotime).

### Reagents

OCT (Tissue Freezing Medium, Sakura, Torrance, CA). Ferristatin II, deferoxamine (DFO), Ferric ammonium citrate (FAC), methyl-β-cyclodextrin (MβCD), nystatin, and LMP agarose were obtained from Sigma-Aldrich (St Louis, MO, USA). Src kinase inhibitor PP2 was bought from SelleckChem (Texas, USA). Protease inhibitor cocktail and Pierce BCA Protein Assay kit (Thermo Scientific). Enhanced chemiluminescence was purchased from New Cell & Molecular Biotech Co., Ltd (China). SABC-POD (rabbit and mouse IgG) kit and peroxidase substrate kit were from BOSTER (Wuhan, China). Protein A/G magnetic beads (B23201, Bimake, USA). Plasmid isolation and gel elution kits were purchased from Axygen Biosciences (Union City, CA, USA). DNA marker was purchased from Novoprotein (DM028-01A). DNA polymerase and Taq polymerase were purchased from TaKaRa Biotechnology Corporation (Dalian, Japan). Restriction enzymes purchased from New England Biolabs (England). The amount of DNA used in the experiments was quantified using a NanoDrop 2000 spectrophotometer (Thermo Scientific USA). Lenti-X HTX Packaging Mix (Clonetech). X-tremeGENE HP DNA Transfection Reagent (Roche, Switzerland). NP-40 lysis buffer (Beyotime). Phen Green SK diacetate (PG SK) was from Life Technologies (Grand Island, NY). RIPA lysis buffer and SDS-PAGE Sample Loading Buffer (5X) (FcMACS, Nanjing, China). TRIZOL reagent and SYBR Green qPCR Kit (Takara, Dalian, China). HiScript II Q RT SuperMix (Vazyme, Nanjing, China).

### Cell culture

Vero E6 cells (ATCC CRL-1586) were kindly provided by the Veterinary Medicine Research Center of the Da Bei Nong Group [82]. Caco-2 and IPEC-J2 (Guangzhou Jennio Biotech Co, Ltd., China). HEK 293T cells were purchased from ATCC (United States) [83]. Caco-2, and IPEC-J2, Vero and HEK 293T cell lines were cultured in Dulbecco’s Modified Eagle’s Medium (DMEM from Life Technologies). All cells were supplemented with 10% fetal bovine serum (FBS, GIBCO), 16 mM HEPES (Life Technologies), and 100 μg/ml penicillin/streptomycin (Invitrogen) in a humidified atmosphere containing 5% CO_2_ at 37°C. Cells were routinely seeded at a density of 2 × 10^5^/mL in 25 cm^2^ plastic tissue culture flasks (Corning) and passaged every 3–4 days for a maximum of 30 passages and regularly tested for mycoplasma contamination.

### Virus strains

The wild-type PEDV strain ZJ was obtained from the intestinal contents of a 2-day-old diarrheic piglet on a farm in Jiangsu in 2012 and propagated in Vero cells, and this strain clustered with the emerging virulent strain based on phylogenetic analysis [28,82].

### Animal experiments

All animal studies were approved by the Institutional Animal Care and Use Committee of Nanjing Agricultural University, and followed the National Institutes of Health guidelines for the performance of animal experiments. Caesarian-derived and colostrum-deprived Xinhuai piglets were obtained from a swine herd at the Jiangsu Academy of Agricultural Science (JAAS). The swine herd was seronegative for antibodies against PEDV, PRRSV, PRCV, TGEV, and PCV2. For the PEDV infection experiment, six newborn piglets were divided into two groups. Each experimental group of pigs was kept in a separate incubator and artificially fed 10 ml of milk every 3 hours. At 6 hours post born, piglets of PEDV-infected group were inoculated with PEDV after feeding. In parallel, the uninfected control piglets were orally administered the same volume of PBS. Piglets that died in the PEDV group were immediately necropsied and intestinal samples collected. All piglets were sacrificed at 48 hours post infection (h p.i.) and intestinal samples collected. The tissue of the small intestine was examined by immunohistochemical methods and western blotting was used to detect PEDV and TfR1.

### Ligated loop experiments

Three newborn piglets were anesthetized with pentobarbital sodium at a dose rate of 20 mg/kg body weight, and a midline incision was made just anterior to the navel [84]. Jejunum segments (2 cm/segment) were injected with PEDV (10^7^ PFU/ml, 0.2 ml/segment). 1 hour post injection, intestines were removed, embedded in OCT, and cut into 8 μm sections for immunofluorescence as described below.

Jejunum segments (2 cm/segment) from three newborn piglets were injected with either PBS, FAC, DFO (0.2 ml/segment), or jejunum segments (2 cm/segment) from another three newborn piglets were injected with either PBS, DMSO, Ferristatin II (0.2 ml/segment). 3 h post injection, intestines were removed, embedded in paraffin and cut into 4 μm section for immunohistochemical staining as described below, or stored at −80°C until used for detecting the expression of TfR1 by western blotting as described below.

Another six newborn piglets were subjected to intestinal ligation with the same treatments as described above (PBS, FAC, DFO, DMSO, or Ferristatin II, 0.2 ml/segment) for 3 h, jejunum segments (2 cm/segment) were injected with PEDV (10^7^ PFU/ml, 0.2 ml/segment). After another 6 h, intestines were removed, embedded in paraffin, and cut into 4 μm section for immunohistochemical staining or stored at −80°C until used for detecting the expression of TfR1 by western blotting. During the procedures, piglets were kept warm on a 37°C warming pad.

### PEDV protection experiment

For the PEDV protection experiment, nine newborn piglets without suckling were randomly divided without concern for sex into three groups: PBS, FAC, and DFO, kept in different incubators, and manually fed 10 ml milk every 3 hours. For the preparation of PEDV protection experiment, FAC group piglets were intramuscularly injected with a single injection of FAC at doses of 150 mg iron in 1.5 ml PBS and DFO group piglets were intramuscularly injected with a single injection of DFO at doses of 60 mg in 1.5 ml PBS referring to the previous study [1,85], respectively. PBS group piglets were injected with the same volume of PBS at the same time. Newborn piglets were pre-intramuscular injection at 2 hours after birth and challenged by PEDV after feeding at 5 hours after birth. All piglets were sacrificed and sampled when severe watery diarrhea was observed in the PBS group at 24 h p.i. The jejunum was removed, embedded in paraffin, and cut into 4 μm section for immunohistochemical staining or stored at −80°C until used for detecting the expression of PEDV by western blotting or quantitative reverse transcription-polymerase chain reaction (qRT-PCR). A 10-cm jejunal segment was dissected to isolate primary porcine enterocytes for flow cytometry. 0.3 g of feces were collected to detect the PEDV genome by qRT-PCR using a cotton swab introduced 4 cm into the rectum.

### Primary porcine enterocytes isolation

Enterocytes were prepared from the jejunum as previously described by Wu et al. [86] with minor modifications. Briefly, the jejunum was thoroughly rinsed with ice-cold sterile saline to remove its luminal contents and all visible fat and cut longitudinally. The lumen was flushed once again three times with Dulbecco's Phosphate Buffered Saline (DPBS) containing 20 mM HEPES (pH 7.4) and 5 mM EDTA (disodium). Then, the tissue was cut into small pieces (approximately 2–3 mm^3^) and transferred to a 50 ml tube containing 40 ml DPBS containing 20 mM HEPES (pH 7.4) and 5 mM EDTA (disodium). The tissue pieces were gently washed by aspiration using a 10 ml pipette and rinsed three times. Then the tissue pieces were incubated with DPBS containing 20 mM HEPES (pH 7.4) and 5 mM EDTA (disodium) at 37°C in a shaking water bath (70 oscillations/min) for 30 min. At the end of the incubation, the tissue pieces were gently patted with fingertips for 1 min, and the cell suspension was filtered through a metal net with a pore size of 200 μm and centrifuged at 300 g at 4°C for 5 min, then washed three times with DPBS containing 20 mM HEPES (pH 7.4) but no EDTA, and resuspended in this buffer. The viability of the prepared enterocytes was assessed by trypan blue exclusion and the resulting cells were used for flow cytometry.

### Immunohistochemical detection of PEDV and TfR1

Paraffin embedded intestinal segments were serially sectioned into 4 μm sections. Paraffin sections were dewaxed in xylene then rehydrated in decreasing concentrations of ethanol. For immunohistochemical staining, antigen retrieval was performed for 30 min with citrate buffer at pH 6.0 in a Decloaking Chamber at 95°C. Slides were blocked with 5% normal goat serum then incubated with primary antibody overnight at 4°C in a humidified chamber. For negative controls, sections were incubated in buffer without primary antibody. The SABC-POD (rabbit or mouse IgG) kit and peroxidase substrate kit were used for amplification and visualization of signal, respectively. Following each incubation step, slices were washed 4 times with fresh PBS-Tween. The sections were visualized using an Olympus BH-2 microscope (40×).

### *In vitro* virus infection

Cells were seeded with or without treatment for the indicated times, inoculated with PEDV at a MOI of 1 for 1 h at 4°C, then washed three times with cold phosphate-buffered saline (PBS) to remove the unattached virus. The cells were shifted to a humidified 5% CO_2_ incubator at 37°C and maintained in DMEM supplemented with 2% FBS and 100 μg/ml penicillin/streptomycin. Infected cells and supernatants were harvested after the incubation period.

### Indirect immunofluorescence assay

To determine whether TfR1 and PEDV co-localize, IPEC-J2 cells and Vero cells seeded on cover slips in 24-well tissue culture plates and infected with PEDV for 1 h at 4°C, rinsed, then incubated at 37°C. At various times post infection cells were fixed in 4% paraformaldehyde for 10 min, rinsed, then permeabilized with 0.1% Triton X-100 in PBS for 5 min, rinsed again then blocked with 5% bovine serum albumin. Cells were incubated with primary antibodies (1:100) overnight at 4°C. Cells were then rinsed and incubated with fluorochrome-conjugated secondary antibodies (1:200) for 30 min at room temperature, washed again three times with PBS and incubated with 1 ug/ml DAPI for 5 min. Images were captured using a Zeiss LSM710 confocal microscope (Carl Zeiss, Germany) and analyzed using ZEN 2012 (Blue edition) (Carl Zeiss).

### Western blotting

At the indicated times post infection, cells were washed with PBS and lysed in ice-cold cell lysis buffer with protease inhibitor cocktail. Total protein concentration was determined with a Pierce BCA Protein Assay Kit, using the bicinchoninic acid spectrophotometric method. Samples containing equal amounts of protein were separated SDS-PAGE and transferred to a PVDF membrane. The membrane was blocked with 5% nonfat milk in Tris-buffered saline (TBS) containing 0.1% Tween 20, then incubated overnight at 4°C with primary antibodies (1:1000), then rinsed and incubated with the corresponding HRP-conjugated secondary antibodies (1:5000) for 60 min at 37°C. Antibody binding was detected by autoradiography using ECL. Western blotting was quantified by Quantity One (Quantity One 1-D Analysis Software 170–9600, Bio-Rad). The intensity of the bands in terms of density was measured and normalized against GAPDH expression. All data were expressed as means ± SD of three independent experiments.

### Plaque assay

Confluent monolayers of Vero cells in 12-well plates were inoculated with serial ten-fold dilutions of virus suspension and incubated for 1 h at 37°C. The cells were then overlaid with 0.7% low melting point agarose in DMEM containing 2% FBS and incubated about 72 h at 37°C. To visualize plaques, cells were stained with 1% crystal violet in methanol.

### Flow cytometry

Flow cytometry was used to detect PEDV entry into cells as follows: The cells (10^6^ cells) were washed with PBS, then harvested using 0.25% trypsin and washed once with PBS, then resuspended in fixation/permeabilization solution (BD Cytofix/Cytoperm kit, BD Pharmingen) and stained with FITC-conjugated porcine anti-PEDV polyclonal antibody (1:100) to detect intracellular PEDV. After three washes with PBS, acquisition of the fluorescent cells done using a BD FACSVerse (Becton Dickinson) and the data were analyzed by using FlowJo software (Version 10).

To compare the intracellular iron concentration of cells treated or untreated with FAC or DFO, we compared their MFI (mean fluorescence intensity) using the metal sensor Phen Green SK diacetate (PG SK). PG SK chelates intracellular labile iron, which in turn quenches the fluorescence of a fluorescein moiety [87]. Cells were grown in 6-well plates until 80%, washed twice with PBS and incubated with 5 μM PG SK for 10 min at 37°C in the dark or left untreated. Cells were harvested by scraping and centrifuged at 1,000 rpm for 5 min and washed with PBS twice. The cell pellets were suspended in 500 μl PBS and transferred to FACS tubes. Fluorescence was analyzed by flow cytometry (BD FACSVerse) and the data were analyzed by FlowJo software (Version 10).

### RNA isolation and qRT-PCR analysis

For examining virus entry, we added the Trizol to collect the samples infected with PEDV at an MOI of 1 at 37°C for 1 h. Fecal swabs and tissue homogenates were added to tubes containing Trizol and vortexed. Total RNA was extracted by the Trizol reagent, according to the manufacturer’s protocol. RNA was subjected to qRT-PCR, as previously described [88]. Gene expression from three independent experiments was calculated with the comparative Ct method and normalized to the endogenous levels of GAPDH. Primer sequences used for qRT-PCR are listed in the S1 Table.

### Plasmid construction

pLVX-DsRed-Monomer-N-TfR1, pAcGFP1-C-TfR1-Out, and pLVX-shTfR1 are stocks kept in our laboratory. shRNA sequences targeting TfR1 were designed using tools available at http://rnaidesigner. lifetechnologies.com/rnaiexpress/insert.do and BLOCK-iT RNAi Designer. shRNAs were cloned into pLVX-shRNA1 (using EcoRI/BamHI) (Takara, Dalian, China). PEDV Spike (S), Spike 1 (S1) and Spike 2 (S2) were cloned into pCMV-C-HA (using BamHI/XbaI). All primers used in PCR are described in the S2 Table.

### Gene transduction

To produce lentivirus, we transfected the TfR1 expression construct or shTfR1 into HEK 293T cells using the Lenti-X HTX Packaging Mix (plasmids pLP1, pLP2, and VSV-G) and the X-tremeGENE HP DNA Transfection Reagent, following the manufacturer’s instructions. 12 h post transfection, the culture medium was renewed and incubation continued for two and three days. Virus-containing supernatants were collected, filtered (pore size 0.45 μm), and stored at −70°C.

Caco-2 and 293T cells were infected with lentiviral particles (MOI 1) containing the TfR1 expression construct. 24 h post infection the culture medium was refreshed, and incubation continued for 12–24 h to allow for maximum protein expression.

IPEC-J2 cells and Vero cells were transfected with the TfR1 shRNA (shTfR1) and the negative control (shCtrl). To generate stably transformed cells (IPEC-J2-shTfR1 and Vero-shTfR1), lentiviral particles (MOI of 1) were added to the cells in the presence of 8 μg/ml polybrene. After incubation for 8 h, the cell cultures were expanded and maintained for 2 weeks in DMEM with 5 μg/ml puromycin. Surviving cells were maintained in medium supplemented with 2 μg/ml puromycin. Lysates from transduced cells were analyzed by western blotting.

### Immunoprecipitation (IP)

Cells were seeded into 10 cm dishes then infected or mock infected with PEDV. At 1 h post infection, cells were collected on ice using 1ml RIPA lysis buffer containing protease inhibitor cocktail and then centrifuged at 12,000×g for 15 min. To eliminate nonspecific binding to the beads, the supernatants were incubated with 2 μl anti-rabbit IgG and 20 μl fresh protein A/G magnetic beads for 1 h at 4°C with gentle shaking. The beads were removed using a magnetic separator, lysates were then incubated with 1 μg of anti-TfR1 or anti-Src Ab overnight at 4°C on a rocker platform. 20 μl of fresh protein A/G magnetic beads were then added to the mixture, and incubated for 2 h at 4°C on a rocker platform. The TfR1- or Src-enriched magnetic beads were washed with PBS, combined with PAGE loading buffer, and incubated for 10 min at 100°C to release the immunoprecipitated proteins. The proteins were analyzed by western blotting using anti-TfR1 Ab or anti-Src Ab. In parallel, magnetic beads loaded with TfR1 were incubated with purified PEDV (MOI 5) in 1 ml for 5 h at 4°C on a rocker platform. Beads were washed four times with PBS and prepared for western blotting using the same methods. The blot was probed with anti-PEDV-N Ab and anti-TfR1 Ab.

### Co-immunoprecipitation (Co-IP) assay

To identify the TfR1 binding domain, we co-transfected HEK 293T cells (at 80–90% confluence) with plasmids expressing PEDV S-HA and TfR1, PEDV S1-HA and TfR1, PEDV S2-HA and TfR1 (or PEDV S1-HA and TfR1-out). Transfection was conducted using the X-tremeGENE HP DNA Transfection Reagent following the manufacturer’s instructions. After 24 h, cells were lysed in NP-40 lysis buffer containing protease inhibitor cocktail then centrifuged at 12000×g for 10 min. The supernatants were pretreated with protein A/G magnetic beads and IgG (from the same species as the immunoprecipitating antibody) for 1 h at 4°C to eliminate nonspecific binding to the beads. The beads were removed by centrifugation and the supernatant was then incubated with the immunoprecipitating antibody overnight at 4°C on a rotary mixer. Fresh protein A/G magnetic beads were added to the mixture and incubation continued for 3 additional hours at 4°C. The complexes were analyzed by western blotting using antibodies against HA, GFP, and TfR1.

### Expression and purification of His-tagged TfR1-Out recombinant protein

The TfR1-Out recombinant protein was expressed in *Escherichia coli* BL-21 and purified using Ni-NTA resin, following the manufacturer’s protocol as previously described [88]. Expressed 32a protein was used as a control.

### Statistical analysis

Data are presented as means ± standard deviation (SD) from three independent experiments. Statistical analysis was performed using the Statistical Program for Social Sciences (SPSS) 16.0. Differences between control and experimental groups were analyzed using Student’s *t*-test and one-way Analysis of Variance (ANOVA). Differences were considered statistically significant at * 0.01 < p < 0.05, ** p < 0.01.

## Supporting information

S1 Fig Microscopic observations of intestinal sections stained with Perls’ Prussian blue (Magnification, × 40). (**a**) d0 piglets show few blue granules in the intestinal villi. (**b**) d31 piglets show heavy accumulation of blue granules in the intestinal villi indicating considerable iron deposition. (**c**) d31 piglets’ intestinal section pretreated with ammonium oxalate then stained with Perls’ Prussian blue as a negative control without blue patches (gradual reduction of blue patches designates significant removal of iron).
(TIF)

S2 Fig Indirect immunofluorescence of the distribution of TfR1 in d0 piglets and d31 piglets. Sections were stained for confocal microscopy using rabbit anti-TfR1 Ab, followed by Dylight 649-conjugated goat anti-rabbit IgG (red). Nuclei were stained with DAPI (blue). The white arrows mark areas of high TfR1 expression (scale bar = 100 μm). In newborn piglets, TfR1 is more highly expressed in the apical surface of the intestinal villi than in d31 piglets.
(TIF)

S3 Fig Immunohistochemical staining of jejunum sections (Magnification, × 40). (**a**) PEDV uninfected control (PBS) piglets show no PEDV-N antigen-positive cells in the intestinal villi. (**b**) PEDV infected piglets’ jejunum section stained with anti-mouse IgG (from the same species substituted for the primary antibody) as negative control without PEDV-N antigen-positive cells.
(TIF)

S4 Fig Western blot of mock or PEDV (MOI 1) infected Vero cells harvested at 12, 24, and 48 h p.i. The cell lysates were analyzed by western blotting using anti-TfR1, anti-PEDV-N, and anti-GAPDH antibodies.
(TIF)

S5 Fig Flow cytometry of intracellular iron in IPEC-J2 cells uninfected or infected with PEDV (MOI 1) at 24 h p.i. The fluorescence profile of each sample and the quantitative analysis demonstrate the decrease in intracellular iron with PEDV infection.
(TIF)

S6 Fig The cytopathic effects in Vero cells pre-incubated with anti-TfR1 Ab or anti-pAPN Ab for 1 h at 37°C then subsequently infected with PEDV for 24 h were observed in the microscope (× 20). Less cytopathic effect was observed in cells treated with anti-TfR1 than in cells treated with anti-pAPN, and the result further confirmed that blocking TfR1 instead of pAPN can inhibit cytopathic effects by PEDV infection at 24 h p.i. The black arrows indicate PEDV infection promotes observable cytopathic effect.
(TIF)

S7 Fig Confocal microscopy of IPEC-J2 cells pretreated with nystatin or MβCD then infected with PEDV (MOI 1) for 1 h. Cells were stained with rabbit anti-TfR1 pAb and mouse anti-PEDV N mAb, followed by Dylight 649-conjugated goat anti-rabbit IgG (red) and Dylight 488-conjugated goat anti-mouse IgG (green). Nuclei were stained with DAPI (blue). Acute cholesterol depletion from nystatin and MβCD specifically reduces TfR1 recruitment. The white arrows indicate PEDV infection promotes TfR1 re-localization and clustering (scale bar = 10 μm).
(TIF)

S1 Table Primer sequences used for qRT-PCR. (XLSX)

S2 Table Primer sequences used for plasmid construction. (XLSX)
