# Supplementary figures and images for: Transferrin receptor 1 levels at the cell surface influence the susceptibility of newborn piglets to PEDV infection
Source: PLoS Pathog. 2020 Jul 30;16(7):e1008682. doi: 10.1371/journal.ppat.1008682 (PMC7419007; doi:10.1371/journal.ppat.1008682)

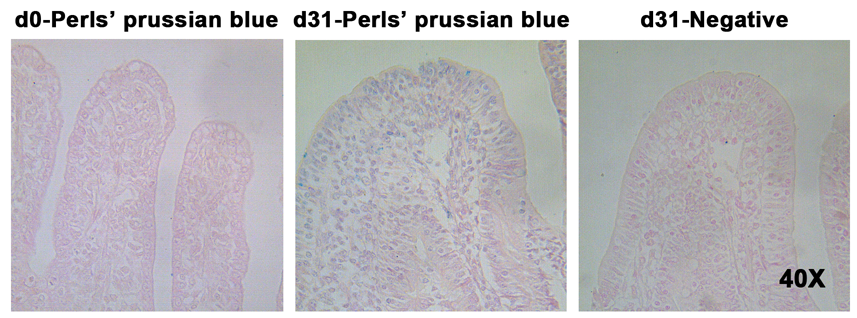

Supplement: S1 Fig — (a) d0 piglets show few blue granules in the intestinal villi. (b) d31 piglets show heavy accumulation of blue granules in the intestinal villi indicating considerable iron deposition. (c) d31 piglets’ intestinal section pretreated with ammonium oxalate then stained with Perls’ Prussian blue as a negative control without blue patches (gradual reduction of blue patches designates significant removal of iron). (TIF) [file ppat.1008682.s001.tif]

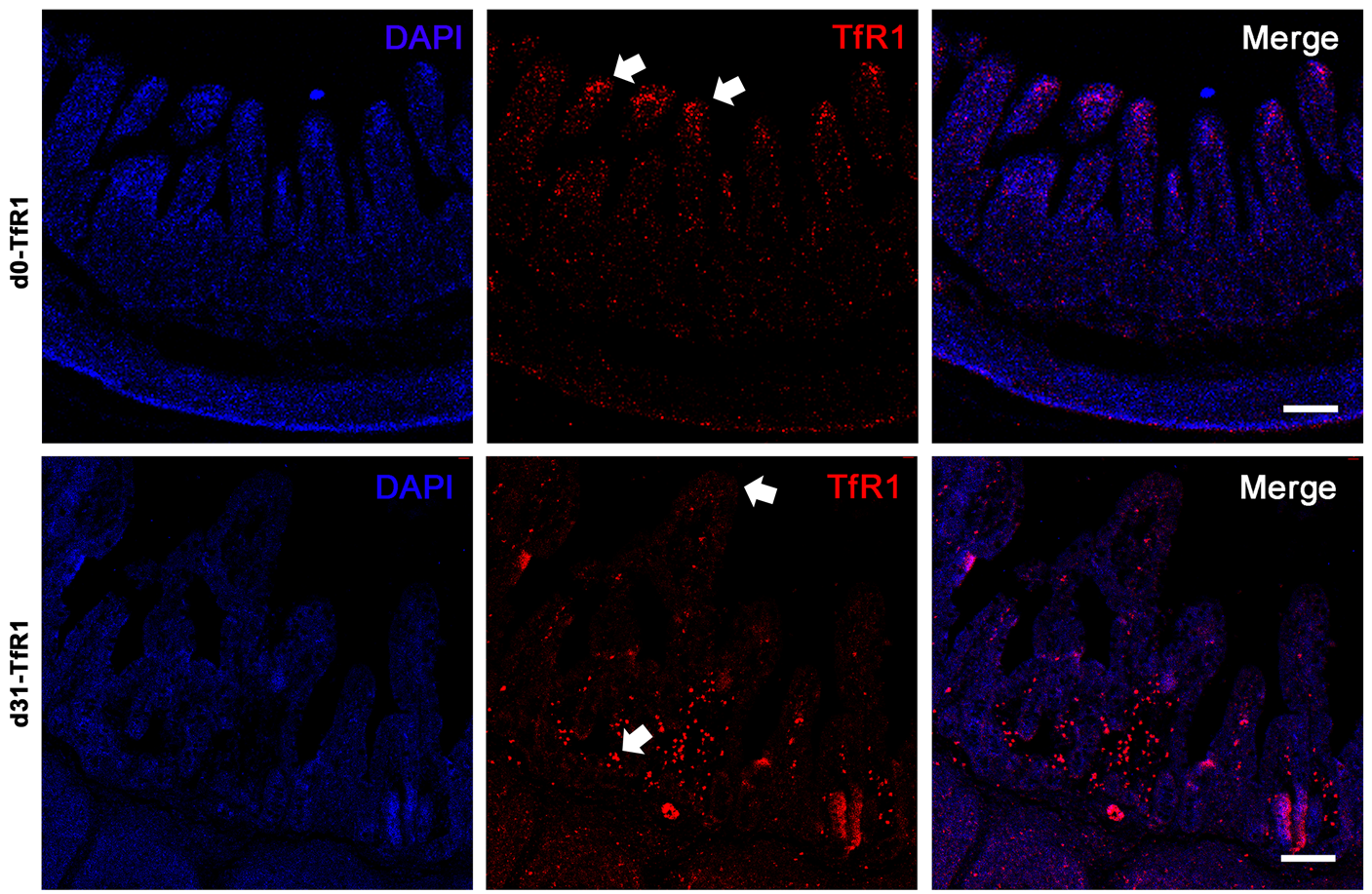

Supplement: S2 Fig — Sections were stained for confocal microscopy using rabbit anti-TfR1 Ab, followed by Dylight 649-conjugated goat anti-rabbit IgG (red). Nuclei were stained with DAPI (blue). The white arrows mark areas of high TfR1 expression (scale bar = 100 μm). In newborn piglets, TfR1 is more highly expressed in the apical surface of the intestinal villi than in d31 piglets. (TIF) [file ppat.1008682.s002.tif]

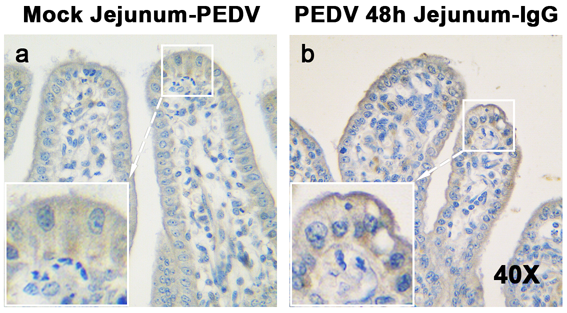

Supplement: S3 Fig — (a) PEDV uninfected control (PBS) piglets show no PEDV-N antigen-positive cells in the intestinal villi. (b) PEDV infected piglets’ jejunum section stained with anti-mouse IgG (from the same species substituted for the primary antibody) as negative control without PEDV-N antigen-positive cells. (TIF) [file ppat.1008682.s003.tif]

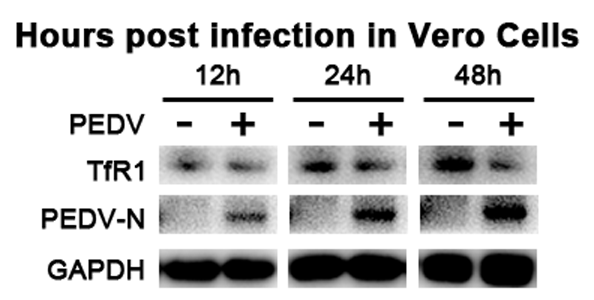

Supplement: S4 Fig — The cell lysates were analyzed by western blotting using anti-TfR1, anti-PEDV-N, and anti-GAPDH antibodies. (TIF) [file ppat.1008682.s004.tif]

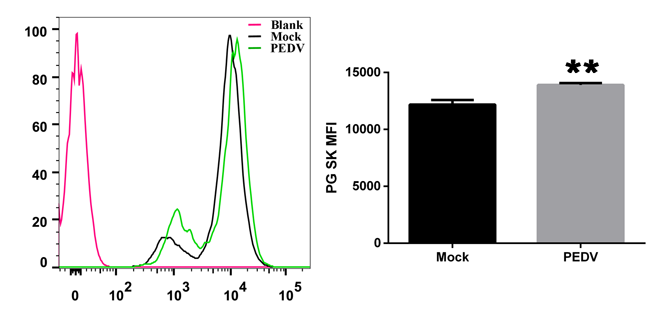

Supplement: S5 Fig — The fluorescence profile of each sample and the quantitative analysis demonstrate the decrease in intracellular iron with PEDV infection. (TIF) [file ppat.1008682.s005.tif]

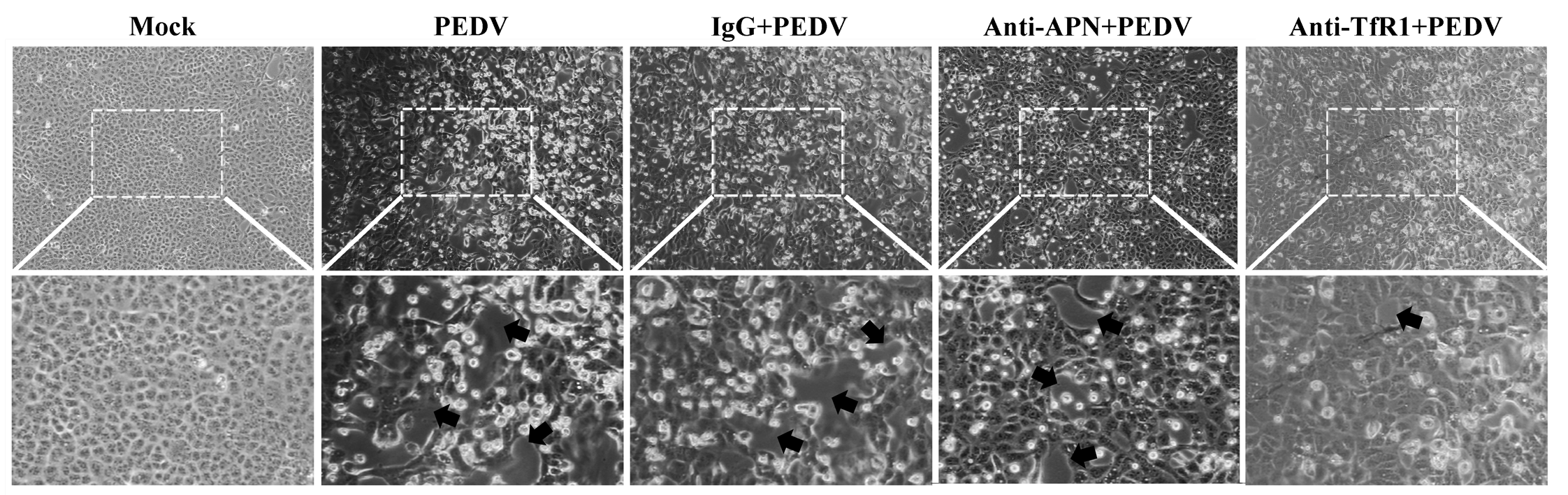

Supplement: S6 Fig — Less cytopathic effect was observed in cells treated with anti-TfR1 than in cells treated with anti-pAPN, and the result further confirmed that blocking TfR1 instead of pAPN can inhibit cytopathic effects by PEDV infection at 24 h p.i. The black arrows indicate PEDV infection promotes observable cytopathic effect. (TIF) [file ppat.1008682.s006.tif]

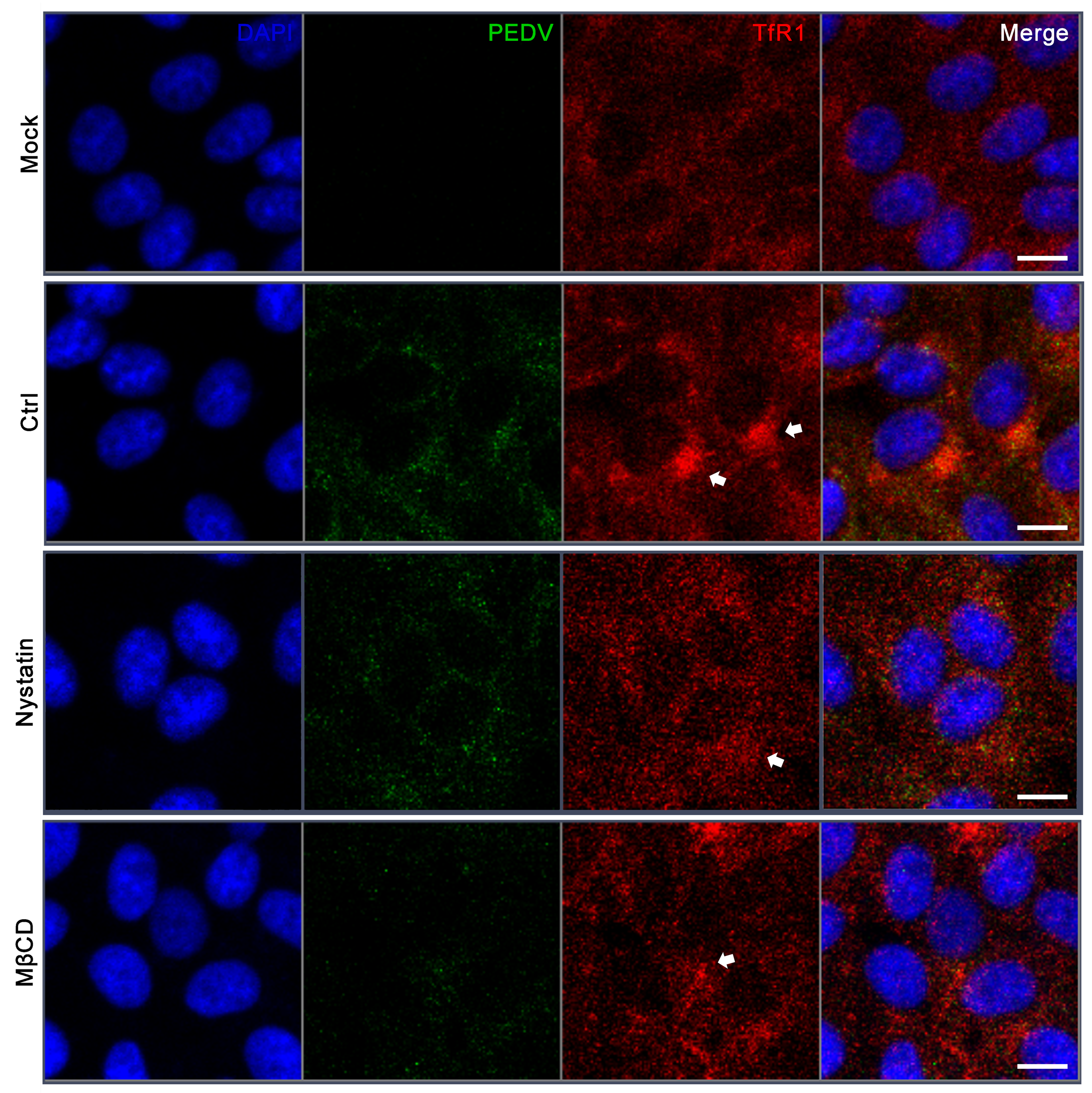

Supplement: S7 Fig — Cells were stained with rabbit anti-TfR1 pAb and mouse anti-PEDV N mAb, followed by Dylight 649-conjugated goat anti-rabbit IgG (red) and Dylight 488-conjugated goat anti-mouse IgG (green). Nuclei were stained with DAPI (blue). Acute cholesterol depletion from nystatin and MβCD specifically reduces TfR1 recruitment. The white arrows indicate PEDV infection promotes TfR1 re-localization and clustering (scale bar = 10 μm). (TIF) [file ppat.1008682.s007.tif]
